# Correction: Measuring political radicalism and extremism in surveys: Three new scales

**DOI:** 10.1371/journal.pone.0345471

**Published:** 2026-03-20

**Authors:** Sebastian Jungkunz, Marc Helbling, Nina Osenbrügge

In Table 1, items LWR3 and LWR4 had incorrect English translations. Please see the correct Table 1 here.

**Table 1:** Question wording for political radicalism and extremism scales.

Item	Question wording
LWR1	A decent living is only possible with socialism.
LWR2	Capitalism is ruining the world.
LWR3	Capitalism ultimately leads to fascism.
LWR4	The (nationality) immigration policy is racist.
LWR5	The persecution of and spying on left-wing system critics by the state and police is increasing.
LWR6	National states should be abolished.
RWR1	We should have the courage to have a strong sense of national consciousness.
RWR2	The (country) has become “too foreign” to a dangerous extent due to all the foreigners here.
RWR3	Jews work more with evil tricks than others in order to get what they want.
RWR4	Jews simply have something special and peculiar about them and do not really fit in with us.
RWR5	Foreigners and asylum seekers are the ruin of (country).
RWR6	Actually, (country) are inherently superior to other people.
RWR7	We should make sure we keep (nationality) pure and prevent nations mixing.
RWR8	The world would be a better place if people from other countries were more like (country).
GEX1	It is better for government leaders to make decisions without consulting anyone.
GEX2	People in government must enforce their authority even if it means violating the rights of some citizens.
GEX3	Under some circumstances, a nondemocratic government can be preferable.
GEX4	A concentration of power in one person guarantees order.
GEX5	The government should close communication media that are critical.

LWR: left-wing radicalism, RWR: right-wing radicalism, GEX: general extremism.
