# Supplementary material for: Measuring political radicalism and extremism in surveys: Three new scales
Source: PLoS One. 2024 May 8;19(5):e0300661. doi: 10.1371/journal.pone.0300661 (PMC11078353; doi:10.1371/journal.pone.0300661)
Supplement: S1 File — (PDF) [file pone.0300661.s001.pdf]

Supplementary Material for "Measuring Political Radicalism and Extremism in Surveys: Three New Scales"

Contents

A Appendix to Main Study 4

B Appendix to Pre-Test 29

C Question Wording 35

The results of our study presented in the main text were subject to intensive pre-testing of a long list of items across a total of four surveys. These include:

1. MTurk: United States, May 2021, n=494, web-survey, convenience sample
2. UADS: Germany, September 2021, n=1,797, web-survey, online-access panel, representative in terms of age, sex, and education
3. Respondi: Germany, January 2022, n=1,520, web-survey, online-access panel, representative in terms of age, sex, and education (see also Appendix B)
4. UADS: Germany, Great Britain, and the Netherlands, n=6,201, web-survey, online-access panel, representative in terms of age, sex, and education (see also Appendix A)

First, we selected all existing scales and items that aim at measuring right- and left-wing radicalism or general extremism. For right-wing extremism items we relied mostly on Mitte-Studien, Stöss (2006), GMF (2008), GMF (2011) and Neu (1997), for left-wing extremism on Stöss (2006), GMF (2004) and Schroeder and Deutz-Schroeder (2015). For general extremism we mainly used items from international surveys that measure democratic attitudes (Arab Barometer, Asian Barometer, Pew Global Attitudes, Mitte Studien, Hoffman 2020, Schulz 2019, Ozer and Bertelson 2018).

We ended up with around 180 items for each form of radicalism and around 100 items for general extremism. Since many scales measure similar concepts and use similar items, we first selected one scale that we found the most comprehensive (Mitte-Studien for right-wing radicalism, Schröder and Deutz-Schröder (2015) for left-wing radicalism) and then added all items from the other scales that were not already included in the first scale. This allowed us to cut down the lists to about 50 items.

For survey 3, we revised our list of items, adding items from related scales in the field and items created by ourselves. Furthermore, we added specific items that we considered

as general extremism, i.e. that is not coupled to any form of ideology. It contained a total of 49 items on left-wing radicalism and extremism, 48 items on right-wing radicalism and extremism, and 32 items on general extremism. Our main work of pre-testing was then carried out in survey 3, as we narrowed down the extensive list of items using exploratory factor analyses. We explain this procedure in greater detail in Appendix B.

Finally, we conducted survey 4 to test the reliability of the items across countries and we further narrowed down the number of items for our scales on left- and right-wing radicalism, and general extremism. We explained this procedure in the main text and provide more details in Appendix A.

We provide a list with all items that we gathered in Appendix C.

A Appendix to Main Study

We conducted a web survey in Germany (N=2,117), Great Britain (N=2,039), and the Netherlands (N=2,045) between 21 June and 13 September 2022 using a recruited Bilendi & respondi online access-panel. The survey was administered in each country's primary language and is representative in terms of sex, age (18 to 69 years), and education.[1] Because extremist attitudes are held by a small percentage of the population (Jungkunz 2022, 2023), we chose countries where the far right and/or far left have recently had electoral successes. This allowed us to find a sizable number of respondents with extremist views and to test whether the scales work in different national contexts equally well.

[1]The survey included an oversampling of Muslims for another reason. As a result, we weighted all models in Mplus (Muthén and Muthén 1998/2017).

Table A.1: Summary Statistics Main Study

	**Mean**	**SD**	**Min**	**Max**
Female	0.50		0	1
Education: high	0.57		0	1
Age	45.08	14.36	16	83
Income	1832.47	1095.41	79.06	6750
Left-Right-Scale	5.93	2.12	1	11

"Education high" refers to *Abitur* and *Fachhochschulreife* in Germany, *A-level*, Scottish *Higher Grades*, *AS level*, *International Baccalaureate*, *Welsh Baccalaureate*, and *Certificate of sixth year studies* in Great Britain, and *MMS*, *HBS*, *HAVO*, *VWO*, and *Gymnasium*, in the Netherlands. Income refers to the OECD equivalized net household income per month. Income was originally measured on a banded scale from one "below 500 Euros" to 10 "4500 Euros or more". We approximated the numerical income by using the mean of each category. For the highest category, we used twice the lower bound for the upper limit, i.e. 9000 Euros. On the left-right self-placement scale, higher values refer to more conservative ratings.

Figure A.1: Distributions of Left-Wing Radical Items by Country

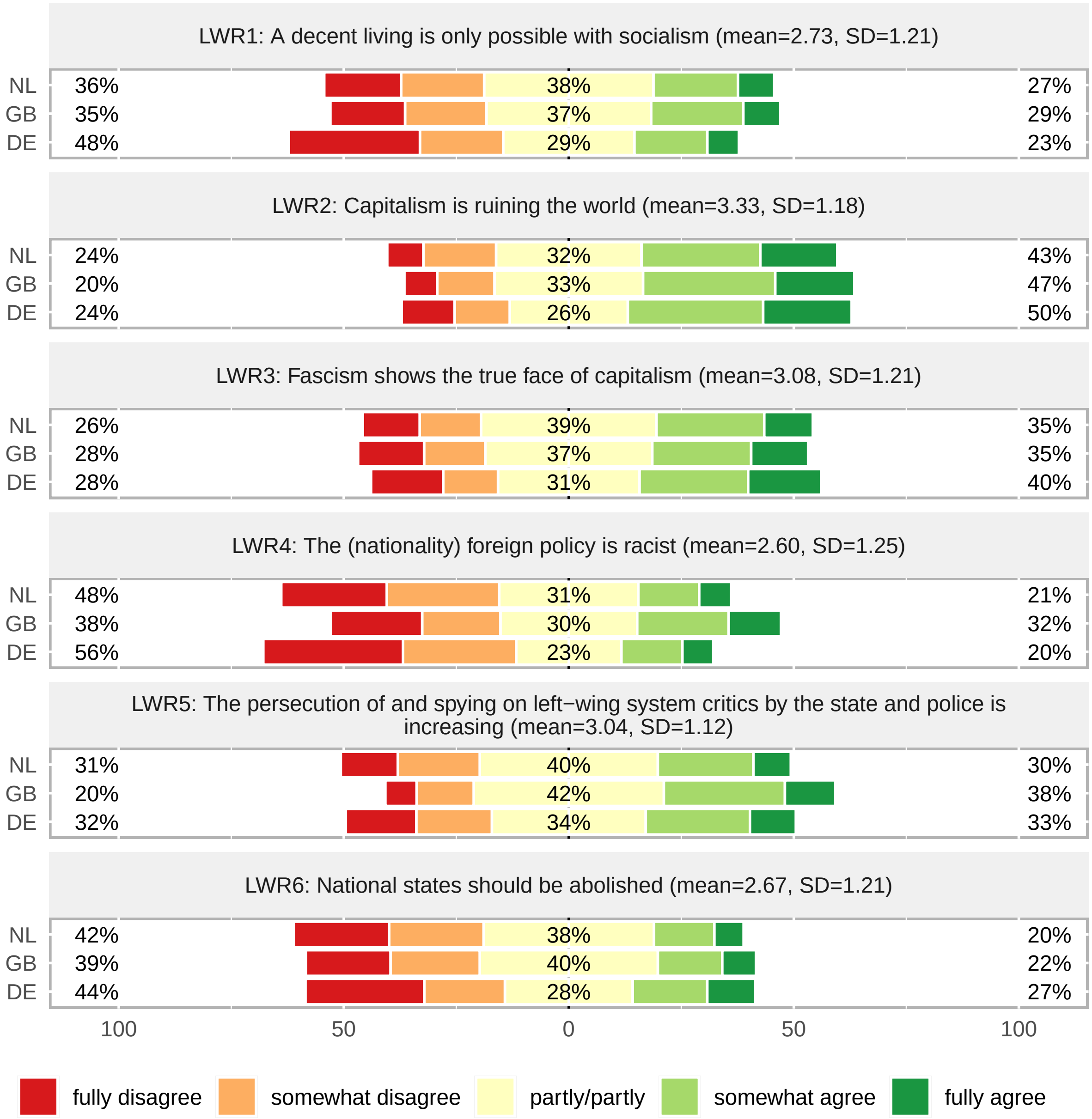

Figure A.2: Distributions of Right-Wing Radical Items by Country

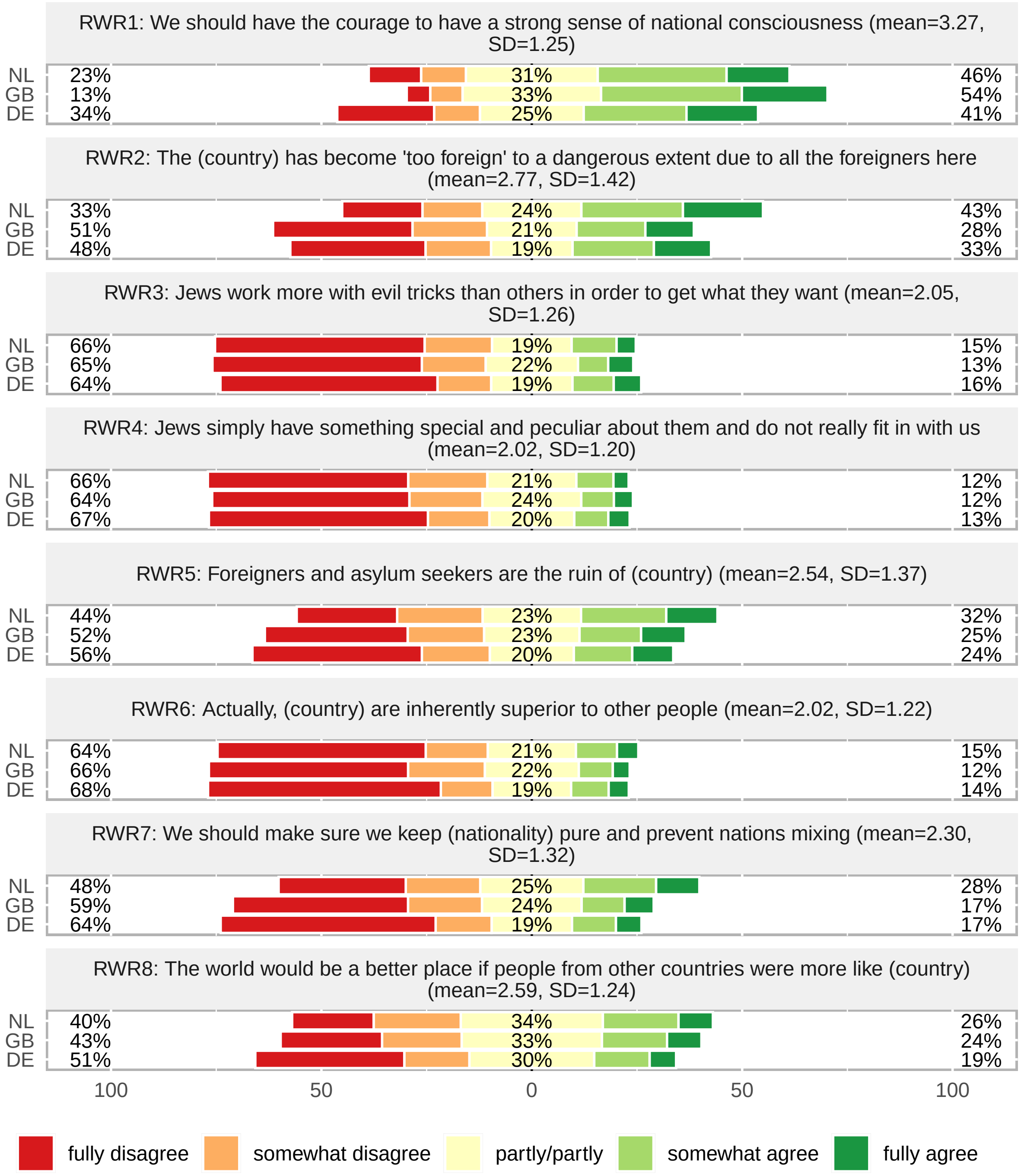

Figure A.3: Distributions of General Extremism Items by Country

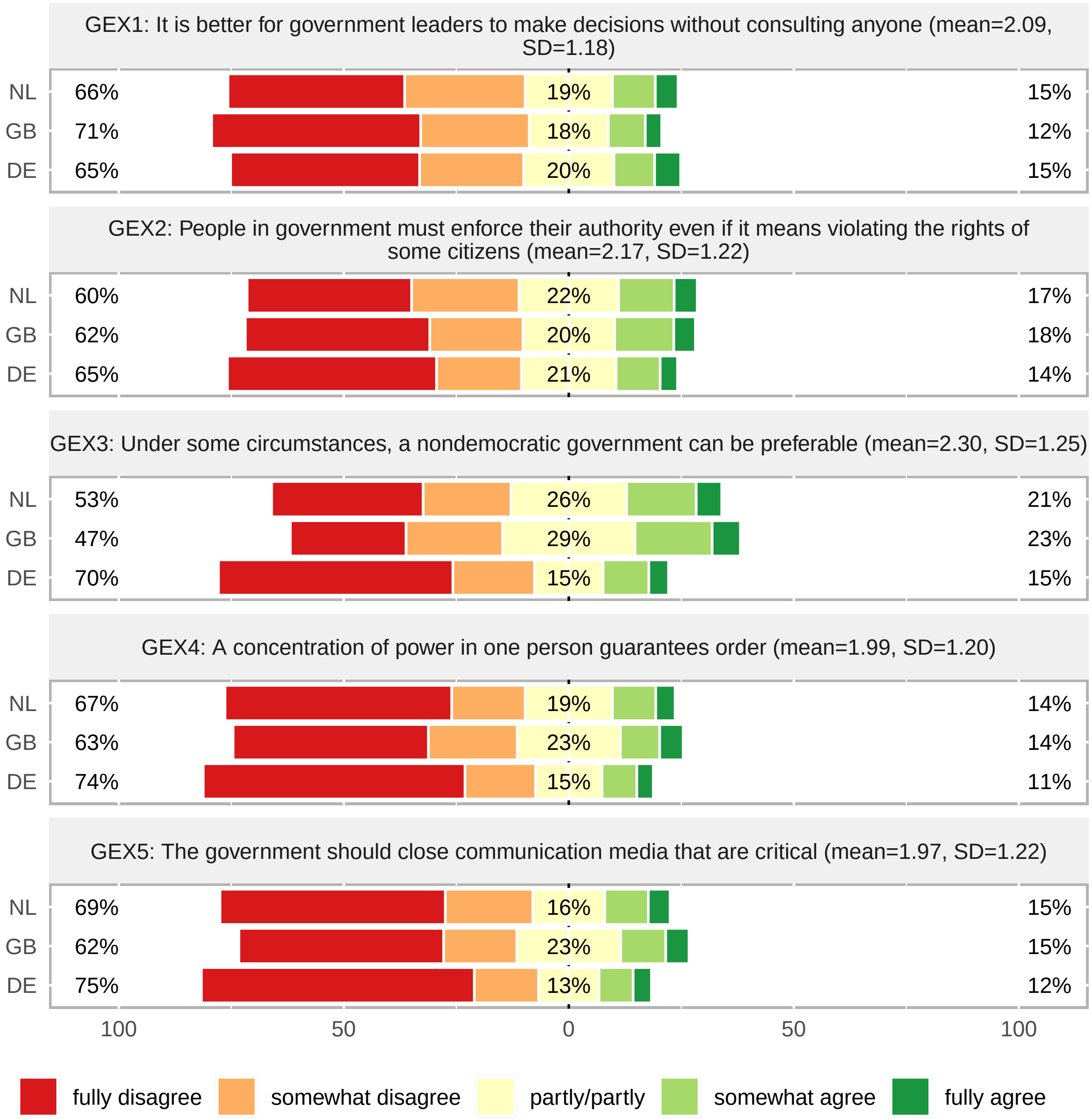

Table A.2: Comparison between Sample and Census Data

	Germany		Great Britain		Netherlands	
	**Census**	**Sample**	**Census**	**Sample**	**Census**	**Sample**
Female	51.2	50.8	51.0	43.8	50.3	55.8
Age: 20-34 years	27.5	26.9	30.7	20.4	30.1	30.9
Age: 35-54 years	39.7	40.4	41.3	49.2	39.6	39.6
Age: 55-69 years	32.8	32.7	28.0	30.3	30.3	29.5
Education: post-secondary (ISCED 5-8)	31.1	33.5	38.1	40.1	34.2	39.9

Numbers in percent may not add up to 100 percent due to rounding. Numbers for age are based on population age 20 to 69 years. Census data for Great Britain is based on UK data (including Northern Ireland). Sources: Federal Statistical Office of Germany (Microcensus 2011), UK Office for National Statistics (Census 2021), Dutch Central Agency for Statistics (Census 2022), United Nations (2023), Statistisches Bundesamt (Destatis) (2022), and UNECE Statistical Database (2023).

Table A.3: Exploratory Factor Analysis Models for Left-Wing Radicalism

Variable	Factor 1	Factor 2	Factor 3	Factor 4	Factor 5	Factor 6	Factor 7
LWR7							
LWR2	0.7824						
LWR8							0.5228
LWR9	0.7769						
LWR37	0.3211		0.5083				
LWR3	0.5404						
LWR10		0.3403		0.4624			
LWR11			0.5351				
LWR4		0.6394					
LWR12		0.6097					
LWR13		0.6905					
LWR14	0.5035					0.3496	
LWR15	0.6199						
LWR1	0.4755	0.3940			0.4385		
LWR16	0.4640				0.4255		
LWR5	0.4445						
LWR17	0.3537	0.3902				0.4068	
LWR6		0.4508		0.3208			
LWR19				0.5091			
LWR20							-0.3126
LWR21		0.5672					0.3352

Loadings represent standardized factor loadings based on Varimax rotation. Loadings <0.3 are blanked.

Table A.4: Exploratory Factor Analysis Models for Right-Wing Radicalism

Variable	Factor 1	Factor 2	Factor 3	Factor 4	Factor 5	Factor 6	Factor 7
RWR9	0.3803			0.3632			
RWR10			0.3985	0.6118			
RWR11			0.3680	0.6179			
RWR1			0.9673				
RWR12	0.5889						
RWR13	0.5565			0.3694			0.3062
RWR14	0.4544		0.3051	0.3996			0.3531
RWR15	0.3520	0.7084					
RWR2		0.7675					
RWR16	0.7782						
RWR3	0.8219						
RWR4	0.8088						
RWR6	0.6449	0.3626					
RWR17	0.4320	0.3107					
RWR22	0.5253	0.4222				0.3080	
RWR7	0.4285	0.6160					
RWR21		0.7287					
RWR5		0.7546					
RWR18					0.5444		
RWR19		0.3976			0.5350		
RWR20					0.4131		
RWR8	0.3495	0.4520					
RWR24							

Loadings represent standardized factor loadings based on Varimax rotation. Loadings <0.3 are blanked.

Table A.5: Exploratory Factor Analysis Models for General Extremism

Variable	Factor 1	Factor 2	Factor 3	Factor 4
GEX6		0.3059	0.6101	
GEX7	0.7530			
GEX9			0.5902	
GEX3	0.6878			
GEX10	0.6822			
GEX11	0.4611			0.3838
GEX12	0.6202			
GEX13	0.4073			0.4269
GEX14	0.5037			0.3493
GEX15		0.4411		
GEX16		0.6626		
GEX17		0.6263		
GEX18		0.5210		
GEX19	0.7296			
GEX1	0.6694			
GEX2	0.6397			
GEX20	0.7655			
GEX4	0.7641			
GEX5	0.7432			
GEX21	0.7889			
GEX22	0.7457			

Loadings represent standardized factor loadings based on Varimax rotation. Loadings <0.3 are blanked.

Table A.6: Confirmatory Factor Analysis Models for Left-Wing Radicalism by Country

	Pooled	DE	GB	NL
LWR4 <- REP	0.586 (0.015)***	0.504 (0.027)***	0.678 (0.023)***	0.551 (0.031)***
LWR5 <- REP	0.637 (0.015)***	0.620 (0.026)***	0.674 (0.024)***	0.604 (0.029)***
LWR6 <- REP	0.460 (0.017)***	0.412 (0.030)***	0.495 (0.028)***	0.482 (0.033)***
LWR2 <- SOC	0.641 (0.014)***	0.685 (0.021)***	0.642 (0.024)***	0.601 (0.027)***
LWR1 <- SOC	0.661 (0.013)***	0.673 (0.020)***	0.704 (0.019)***	0.587 (0.029)***
LWR3 <- SOC	0.682 (0.014)***	0.681 (0.023)***	0.745 (0.019)***	0.637 (0.029)***
N	5555	1960	1765	1830
df	8	8	8	8
Chi^2	95.699	52.684	23.698	29.288
RMSEA	0.044	0.053	0.033	0.038
SRMR	0.022	0.027	0.018	0.024
CFI	0.978	0.970	0.990	0.979

Loadings represent standardized factor loadings of a two-factor model. REP: anti-racism and anti-repression, SOC: socialism and anti-capitalism. $^{*}p < 0.05$, $^{**}p < 0.01$, $^{***}p < 0.001$.

Table A.7: Confirmatory Factor Analysis Models for Right-Wing Radicalism by Country

	Pooled	DE	GB	NL
RWR1 <- NATPR	0.489 (0.014)***	0.585 (0.020)***	0.399 (0.025)***	0.463 (0.030)***
RWR8 <- NATPR	0.708 (0.015)***	0.695 (0.021)***	0.843 (0.027)***	0.566 (0.032)***
RWR2 <- XENO	0.850 (0.008)***	0.879 (0.012)***	0.892 (0.011)***	0.796 (0.018)***
RWR5 <- XENO	0.884 (0.008)***	0.882 (0.011)***	0.896 (0.012)***	0.850 (0.019)***
RWR3 <- ASEM	0.867 (0.009)***	0.885 (0.014)***	0.880 (0.013)***	0.843 (0.017)***
RWR4 <- ASEM	0.896 (0.008)***	0.909 (0.012)***	0.904 (0.012)***	0.872 (0.016)***
RWR6 <- SD	0.768 (0.011)***	0.784 (0.016)***	0.802 (0.017)***	0.721 (0.024)***
RWR7 <- SD	0.741 (0.011)***	0.784 (0.016)***	0.790 (0.016)***	0.668 (0.023)***
N	5673	1980	1817	1876
df	12	12	12	12
Chi^2	303.762	103.264	75.658	195.368
RMSEA	0.065	0.062	0.054	0.090
SRMR	0.030	0.024	0.023	0.048
CFI	0.979	0.982	0.987	0.953

Loadings represent standardized factor loadings of a four-factor model. NAT: nationalism, XENO: xenophobia, ASEM: anti-Semitism, SD: social-Darwinism. $^{*}p < 0.05$, $^{**}p < 0.01$, $^{***}p < 0.001$.

Table A.8: Confirmatory Factor Analysis Models for General Extremism by Country

	Pooled	DE	GB	NL
GEX1 <- GEX	0.626 (0.013)***	0.593 (0.022)***	0.702 (0.020)***	0.652 (0.021)***
GEX5 <- GEX	0.705 (0.012)***	0.622 (0.025)***	0.746 (0.018)***	0.712 (0.020)***
GEX4 <- GEX	0.764 (0.011)***	0.744 (0.021)***	0.777 (0.019)***	0.762 (0.020)***
GEX2 <- GEX	0.711 (0.011)***	0.727 (0.019)***	0.654 (0.022)***	0.716 (0.019)***
GEX3 <- GEX	0.644 (0.012)***	0.697 (0.020)***	0.628 (0.019)***	0.581 (0.022)***
N	5667	1978	1809	1880
df	5	5	5	5
Chi^2	56.700	12.555	24.389	16.463
RMSEA	0.043	0.028	0.046	0.035
SRMR	0.016	0.014	0.018	0.014
CFI	0.989	0.994	0.989	0.992

Loadings represent standardized factor loadings of a one-factor model. *p < 0.05, **p < 0.01, ***p < 0.001.

Table A.9: MGCFA for Left-Wing Radicalism

Model	χ^2	(df)	CFI	RMSEA	(90% CI)	SRMR	Model Comparison	$\Delta\chi^2$	(df)	ΔCFI	ΔRMSEA	ΔSRMR	Decision
M1: Configural Invariance	103.916	(24)	0.981	0.042	(0.034 - 0.051)	0.023							
M2: Metric Invariance	146.807	(32)	0.972	0.044	(0.037 - 0.051)	0.037	M1	42.891	(8)	-0.009	0.002	0.014	Accept
M3: Scalar Invariance	302.856	(40)	0.936	0.060	(0.053 - 0.066)	0.045	M2	156.049	(8)	-0.036	0.016	0.008	Reject
M3a: Partial Scalar Invariance	172.427	(37)	0.967	0.044	(0.038 - 0.051)	0.042	M2	25.620	(5)	-0.005	0.000	0.005	Accept

Δshows differences in values between models (configural vs. metric, metric vs. scalar, and metric vs. scalar (partial)). Item LWR1 was relaxed for GER and item LWR4 for GB and NL.

Table A.10: MGCFA for Right-Wing Radicalism

Model	χ^2	(df)	CFI	RMSEA	(90% CI)	SRMR	Model Comparison	$\Delta\chi^2$	(df)	ΔCFI	ΔRMSEA	ΔSRMR	Decision
M1: Configural Invariance	374.775	(36)	0.976	0.071	(0.064 - 0.077)	0.034							
M2: Metric Invariance	478.626	(44)	0.969	0.072	(0.067 - 0.078)	0.055	M1	103.851	(8)	-0.007	0.001	0.021	Accept
M3: Scalar Invariance	755.775	(52)	0.950	0.085	(0.079 - 0.090)	0.071	M2	277.149	(8)	-0.019	0.013	0.016	Reject
M3a: Partial Scalar Invariance	633.964	(49)	0.959	0.079	(0.074 - 0.085)	0.058	M2	155.338	(5)	-0.010	0.007	0.003	Accept

Δshows differences in values between models (configural vs. metric, metric vs. scalar, and metric vs. scalar (partial)). Item RWR1 was relaxed for GER and NL, and item RWR2 additionally for NL.

Table A.11: MGCFA for General Extremism

Model	χ^2	(df)	CFI	RMSEA	(90% CI)	SRMR	Model Comparison	$\Delta\chi^2$	(df)	ΔCFI	ΔRMSEA	ΔSRMR	Decision
M1: Configural Invariance	34.616	(15)	0.995	0.026	(0.015 - 0.038)	0.013							
M2: Metric Invariance	64.546	(23)	0.990	0.031	(0.022 - 0.040)	0.027	M1	29.930	(8)	-0.005	0.005	0.014	Accept
M3: Scalar Invariance	361.085	(31)	0.923	0.075	(0.068 - 0.082)	0.062	M2	296.539	(8)	-0.067	0.044	0.035	Reject
M3a: Partial Scalar Invariance	92.869	(27)	0.985	0.036	(0.028 - 0.044)	0.033	M2	28.323	(4)	-0.005	0.005	0.006	Accept

Δshows differences in values between models (configural vs. metric, metric vs. scalar, and metric vs. scalar (partial)). Items GEX3, GEX4, and GEX5 were relaxed for GER and item GEX1 for GB.

Figure A.4: Information Curves for Left-Wing Radicalism Scale by Country

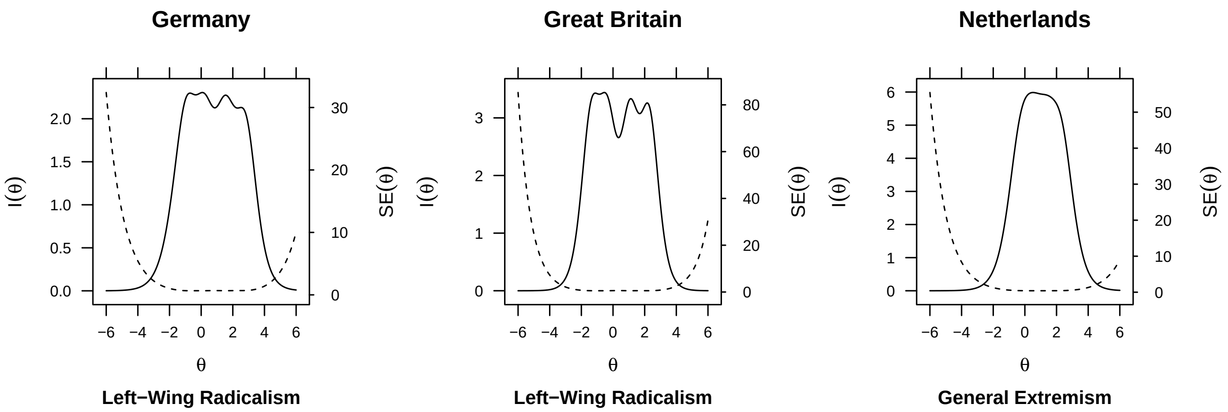

Based on pooled data. Information (solid lines) and SE curves (dashed lines) for radicalism and extremism scales. Higher values represent more radical and extremist attitudes.

Figure A.5: Information Curves for Left-Wing Radicalism Scale by Country

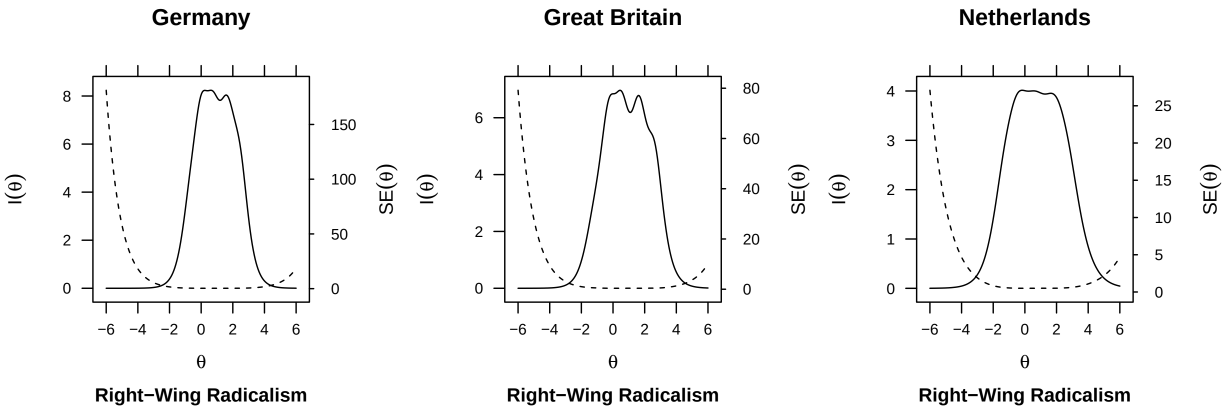

Based on pooled data. Information (solid lines) and SE curves (dashed lines) for radicalism and extremism scales. Higher values represent more radical and extremist attitudes.

Figure A.6: Information Curves for Left-Wing Radicalism Scale by Country

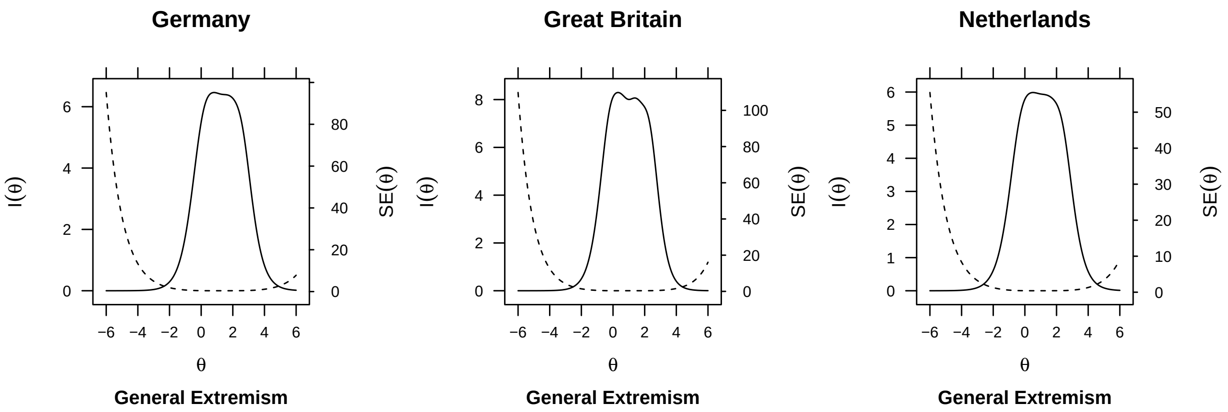

Based on pooled data. Information (solid lines) and SE curves (dashed lines) for radicalism and extremism scales. Higher values represent more radical and extremist attitudes.

Table A.12: Correlations between Radical and Extremist Attitudes and Related Constructs (Germany)

	LWR	RWR	GEX
Authoritarianism personality traits	0.069	0.395	0.349
Conspiracy beliefs	0.417	0.414	0.248
Political detachment	0.330	0.375	0.193
Political violence justification: general	0.310	0.538	0.587
Political violence justification: specific	0.372	0.486	0.623
Idea of democracy	-0.331	-0.403	-0.467
Left-right scale	-0.209	0.370	0.158

LWR: left-wing radicalism, RWR: right-wing radicalism, GEX: general extremism. Higher values on the left-right self-placement scale represent a more conservative, right-wing oriented placement.

Table A.13: Correlations between Radical and Extremist Attitudes and Related Constructs (Great Britain)

	LWR	RWR	GEX
Authoritarianism personality traits	-0.010	0.278	0.344
Conspiracy beliefs	0.381	0.319	0.084
Political detachment	0.356	0.270	-0.017
Political violence justification: general	0.317	0.510	0.522
Political violence justification: specific	0.333	0.481	0.590
Idea of democracy	-0.269	-0.351	-0.327
Left-right scale	-0.206	0.391	0.335

LWR: left-wing radicalism, RWR: right-wing radicalism, GEX: general extremism. Higher values on the left-right self-placement scale represent a more conservative, right-wing oriented placement.

Table A.14: Correlations between Radical and Extremist Attitudes and Related Constructs (Netherlands)

	LWR	RWR	GEX
Authoritarianism personality traits	-0.027	0.007	-0.044
none significant Conspiracy beliefs	0.350	0.351	0.197
Political detachment	0.408	0.368	0.226
Political violence justification: general	0.329	0.503	0.564
Political violence justification: specific	0.308	0.470	0.523
Idea of democracy	-0.264	-0.396	-0.423
Left-right scale	-0.134	0.363	0.201

LWR: left-wing radicalism, RWR: right-wing radicalism, GEX: general extremism. Higher values on the left-right self-placement scale represent a more conservative, right-wing oriented placement.

Figure A.7: Propensity to Vote for Far Left and Far Right Parties by Radicalism and Extremism Scales (Germany)

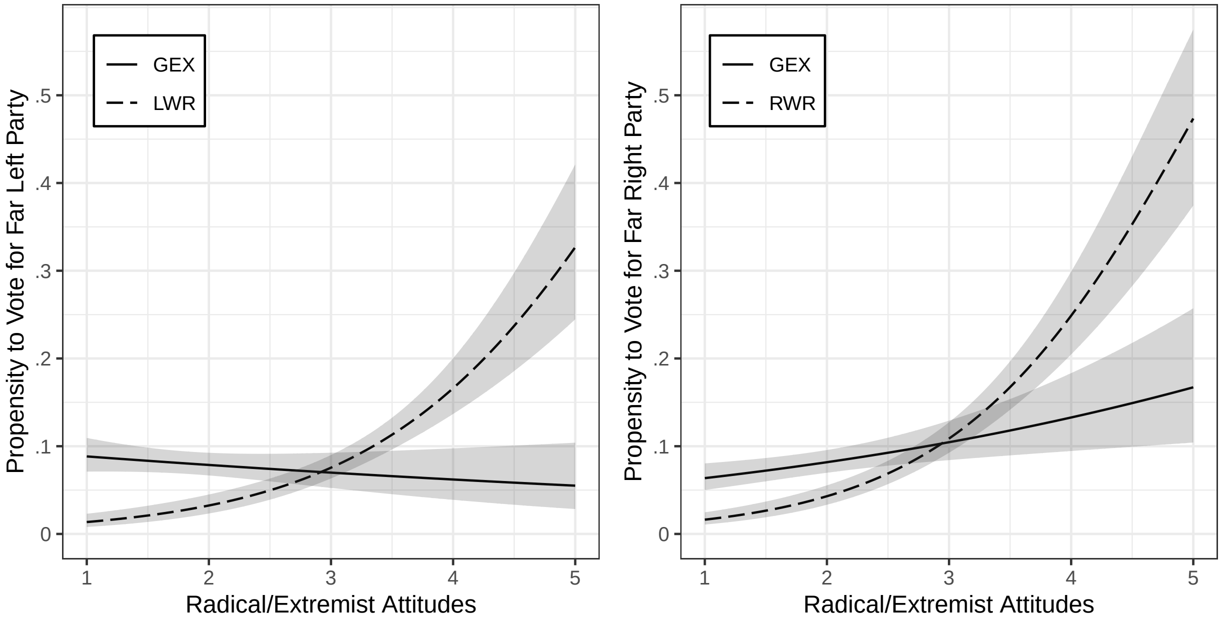

Based on logistic regressions with 95% confidence intervals. LWR: left-wing radicalism, RWR: right-wing radicalism, GEX: general extremism. Higher values represent more radical and extremist attitudes. Far left party includes the Left Party (*Die Linke*), far right party includes the AfD.

Figure A.8: Propensity to Vote for Far Right Parties by Radicalism and Extremism Scales (Great Britain)

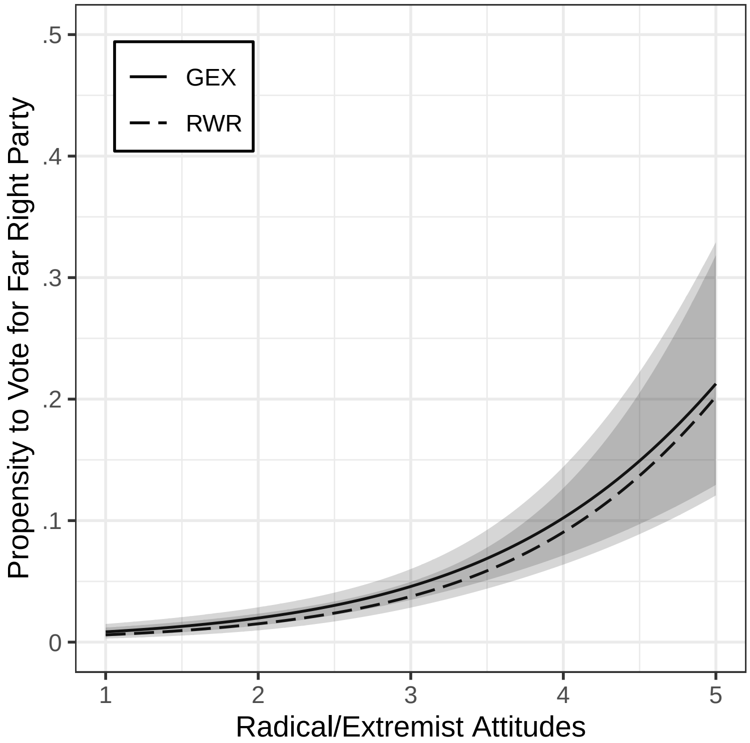

Based on logistic regressions with 95% confidence intervals. RWR: right-wing radicalism, GEX: general extremism. Higher values represent more radical and extremist attitudes. Far right party includes the UKIP.

Figure A.9: Propensity to Vote for Far Left and Far Right Parties by Radicalism and Extremism Scales (Netherlands)

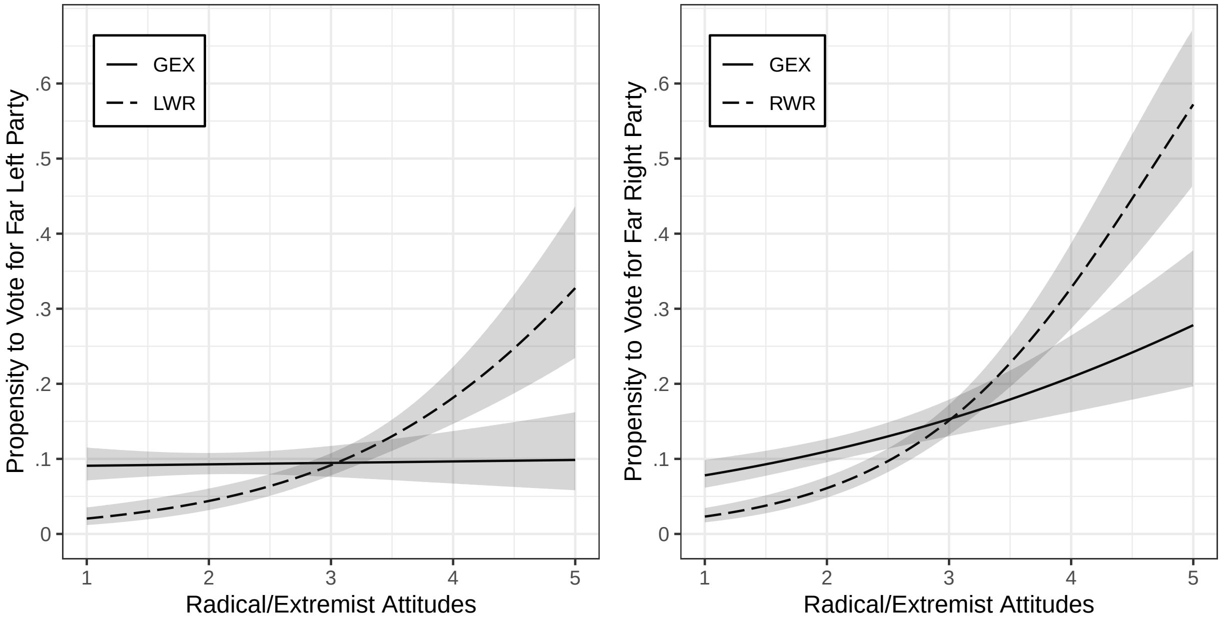

Based on logistic regressions with 95% confidence intervals. LWR: left-wing radicalism, RWR: right-wing radicalism, GEX: general extremism. Higher values represent more radical and extremist attitudes. Far left party includes the Socialistische Partij, far right party includes the PVV and FvD.

Figure A.10: Distributions of Left- and Right-Wing Extremist Attitudes by Country (minimum aggregation method)

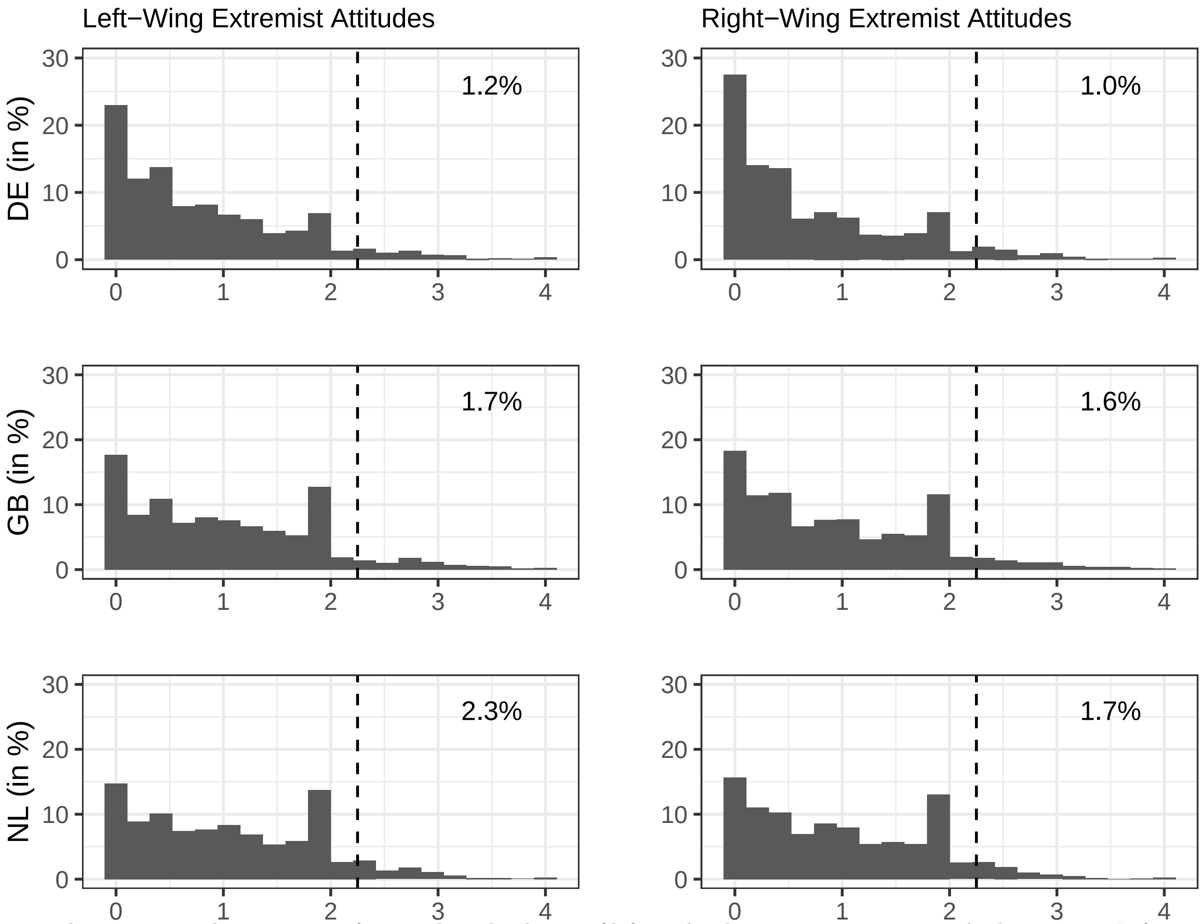

Shown are histograms with percentage of respondents by degree of left- and right-wing extremist attitudes by country. Left-wing extremist attitudes are constructed based on the minimum value across the left-wing radicalism scale and the general extremism scale. Right-wing extremist attitudes are constructed based on the minimum value across the right-wing radicalism scale and the general extremism scale. Higher values indicate stronger extremist attitudes. Numbers in the plot area refer to the share of respondents that on average at least "agreed" with all items.

Figure A.11: Distributions of Left- and Right-Wing Extremist Attitudes by Country (additive aggregation method)

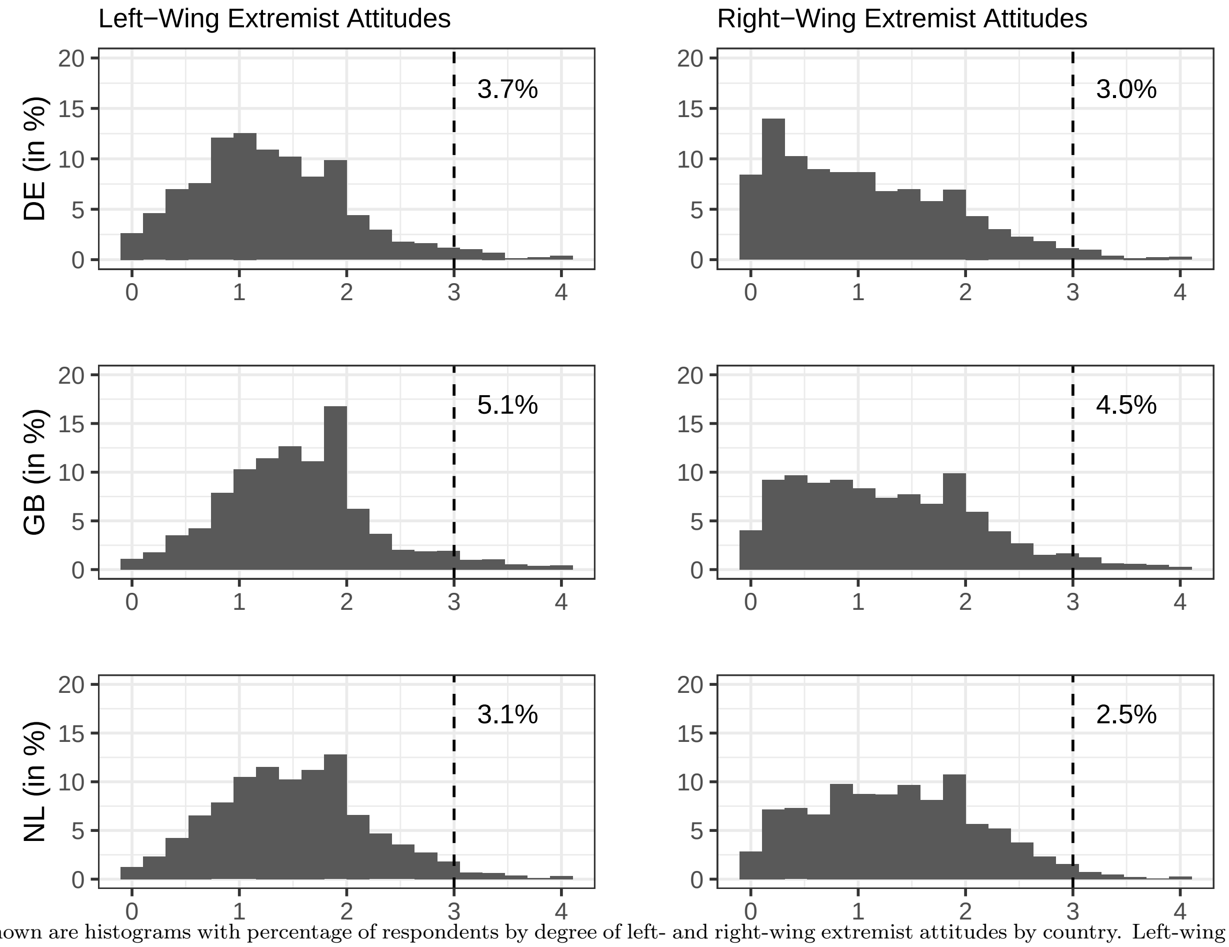

Shown are histograms with percentage of respondents by degree of left- and right-wing extremist attitudes by country. Left-wing extremist attitudes are constructed based on the minimum value across the left-wing radicalism scale and the general extremism scale. Right-wing extremist attitudes are constructed based on the minimum value across the right-wing radicalism scale and the general extremism scale. Higher values indicate stronger extremist attitudes. Numbers in the plot area refer to the share of respondents that on average at least "agreed" with all items.

Figure A.12: Correlations between left-wing radicalism and extremism indices

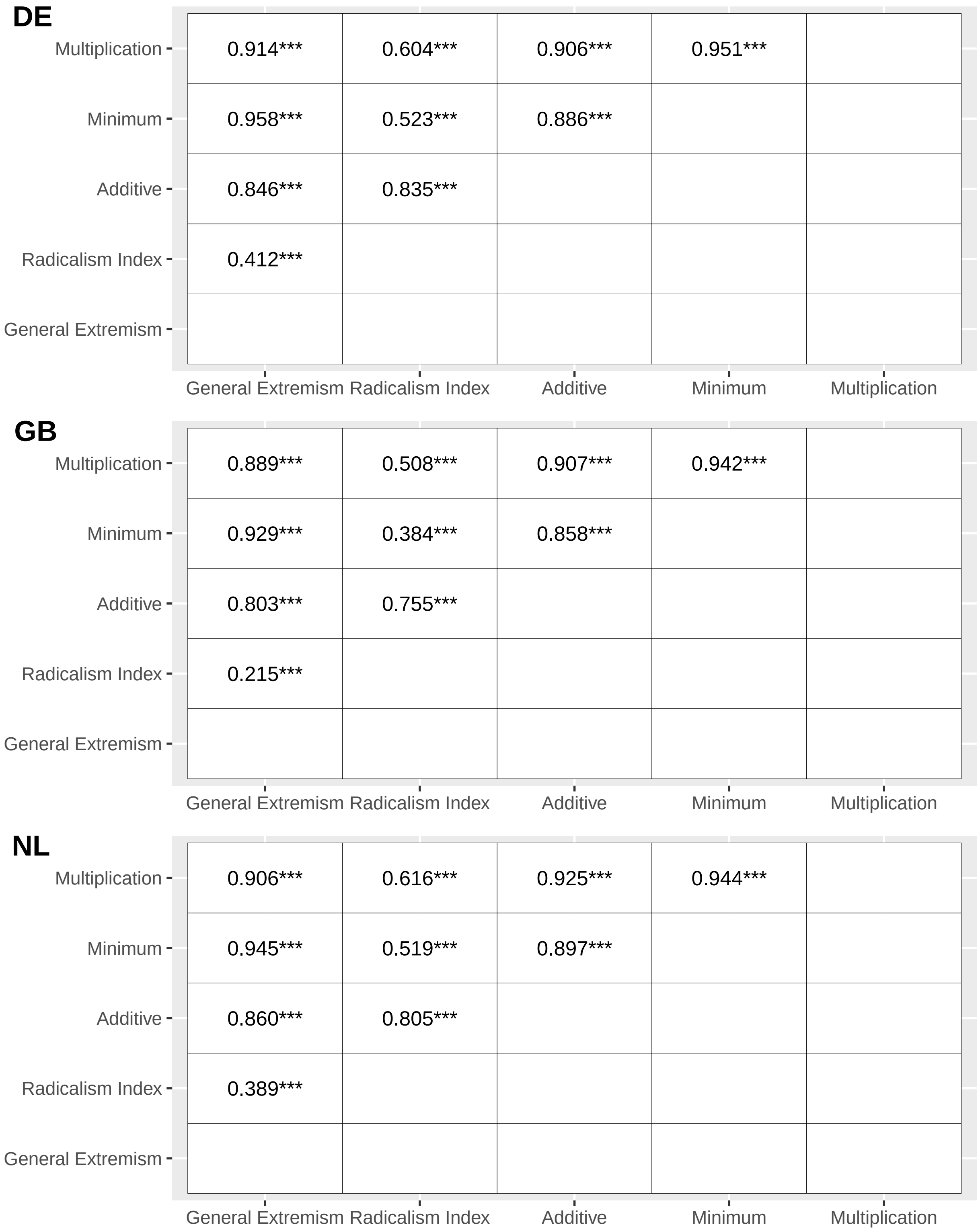

Shown are correlations between indices of left-wing radicalism, general extremism, and three methods of aggregation methods of left-wing extremism by country. $^{***}p<0.001$; $^{**}p<0.01$; $^{*}p<0.05$.

Figure A.13: Correlations between right-wing radicalism and extremism indices

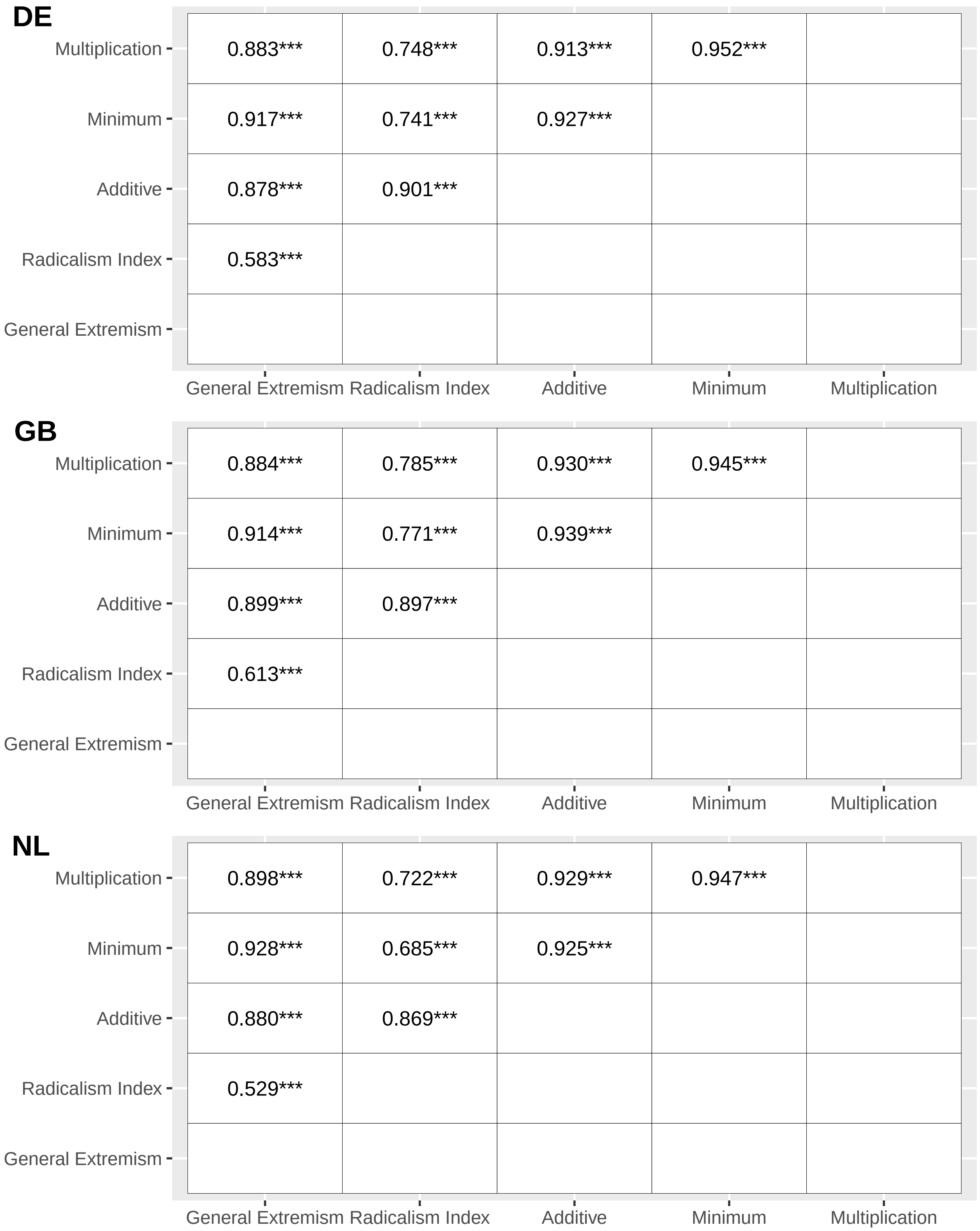

Shown are correlations between indices of right-wing radicalism, general extremism, and three methods of aggregation methods of right-wing extremism by country. ***p<0.001; **p<0.01; *p<0.05.

Figure A.14: Missing data by indicator

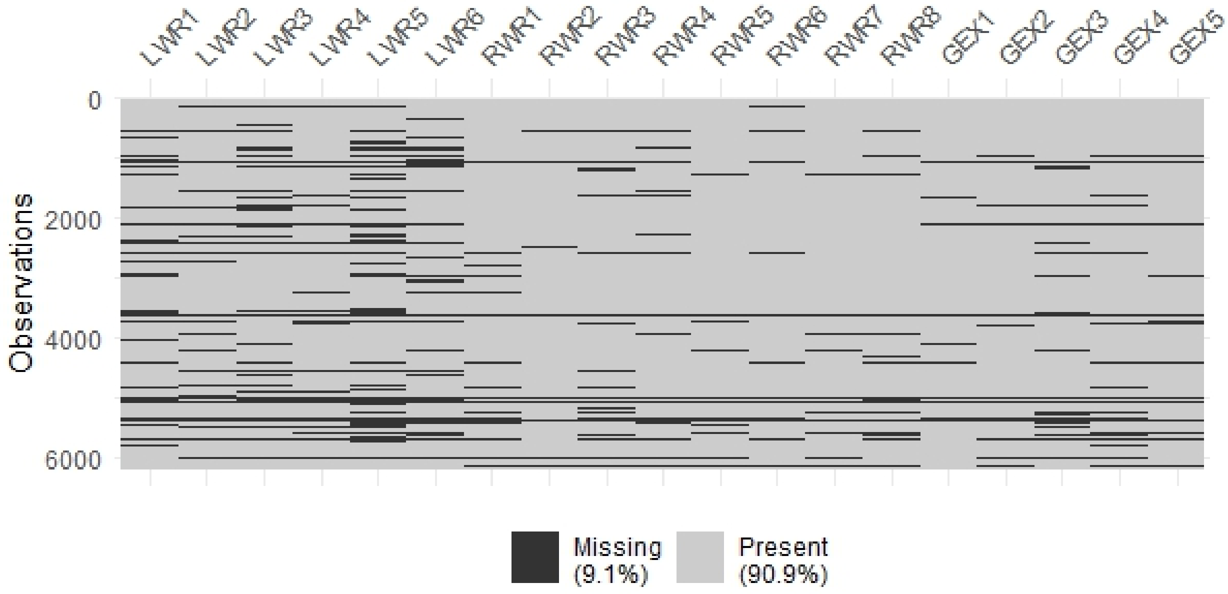

Missing and non-missing data by items.

B Appendix to Pre-Test

The aim of this survey was to test items that could measure the concept of general extremism through explicit items (as we had not done so in previous surveys). Furthermore, we revised the list of items on left-wing and right-wing radicalism. This survey was conducted by respondi using an online access-panel in Germany in January 2022 (n=1,520). Summary statistics can be found in Table B.1. Its main components included:

- A list of items to measure general extremism: Arab Barometer, Asian Barometer, Pew Global Attitudes, the ICCS 2016 Latin American student questionnaire (Schulz et al. 2018), the *Mitte Studien* (Decker, Kiess, and Brähler 2022), Hoffman (2020), and Ozer and Bertelsen (2018)

- A list of items to measure right-wing radicalism and extremism: the *Mitte Studien* (Decker, Kiess, and Brähler 2022), Stöss et al. (2004), GMF Studies in 2004, 2008 and 2011 (Heitmeyer et al. 2013a,b,c), Neu (2009)

- A list of items to measure left-wing radicalism and extremism: Stöss et al. (2004), Falter et al. (2012), GMF Study 2004 (Heitmeyer et al. 2013a), Schroeder and Deutz-Schroeder (2015), Schumann (2001), Baier and Pfeiffer (2011)

- A list of items to measure religious extremism: Altemeyer and Hunsberger (2004), Glazier (2015)

- A number of questions trying to gauge support for political violence and related constructs: Bruder et al. (2013), Cherney et al. (2018), Isenhardt et al. (2021), Kalmoe and Mason (2019), and Ozer and Bertelsen (2018)

We conducted factor analyses on items for general extremism, right-wing radicalism and left-wing radicalism. The results are provided below. While researchers generally seek to identify items with high factor loadings, we also tried to add a substantial conceptual breadth

to our scales. This is important, as the long list of items that we gathered in total might otherwise collapse down to some specific items that correlate strongly, but reflect only certain nuances of the overall concepts of political radicalism and extremism. Thus, there may be instances where we decided to keep an item for the main survey which had a somewhat lower loading but provided greater conceptual breadth to a potential scale of political radicalism and extremism.

Table B.1: Summary Statistics Respondi Pre-Test

	**Mean**	**SD**	**Min**	**Max**
Female	0.50		0	1
Education:				
- none	0.02		0	1
- Hauptschule	0.26		0	1
- Realschule	0.28		0	1
- Polytechnische Oberschule (former GDR)	0.08		0	1
- Abitur	0.37		0	1
Age	45.30	14.33	18	69
Income	1948.05	1107.15	102.06	6750
Left-Right-Scale	4.73	1.80	0	10

"Education high" refers to *Abitur* and *Fachhochschulreife*. Income refers to the OECD equivalized net household income per month. Income was originally measured on a banded scale from one "below 500 Euros" to 10 "4500 Euros or more". We approximated the numerical income by using the mean of each category. For the highest category, we used twice the lower bound for the upper limit, i.e. 9000 Euros. On the left-right self-placement scale, higher values refer to more conservative ratings.

Table B.2: Exploratory Factor Analysis Models for Left-Wing Radicalism

Variable	Factor 1	Factor 2	Factor 3	Factor 4	Uniqueness
LWR7	0.5468	0.3128			0.5484
LWR22	0.5938				0.6034
LWR23	0.6991				0.3866
LWR24	0.6783				0.5096
LWR25					0.9033
LWR26			0.3216		0.8330
LWR27	0.4771				0.7287
LWR2	0.7117				0.3947
LWR28	0.3555	0.5032			0.5235
LWR29	0.4635	0.4310			0.5498
LWR8				0.7493	0.3802
LWR30	0.3785				0.7626
LWR31	0.4350	0.4360			0.5909
LWR9	0.7106				0.4013
LWR32			0.6328		0.5119
LWR33	0.6709				0.4135
LWR34	0.6631				0.4623
LWR35	0.5826				0.5887
LWR36			0.3958		0.6864
LWR37			0.6790		0.5086
LWR3	0.6003		0.3144		0.4768
LWR10		0.4408	0.4751		0.5522
LWR11			0.6492		0.5198
LWR4		0.6158			0.5194
LWR12		0.6429			0.5086
LWR13		0.7434			0.4306
LWR99	0.7920				0.3686
LWR14	0.8200				0.3233
LWR38	0.6339				0.5274
LWR15	0.6613				0.4122
LWR39	0.6137				0.4763
LWR40	0.6060		-0.3313		0.5002
LWR1	0.4417	0.5538			0.4891
LWR16	0.3987				0.6934
LWR41			0.4831		0.7036
LWR5	0.4628		0.3521		0.5946
LWR42		0.5891			0.5129
LWR17	0.6806				0.4434
LWR43	0.6899				0.4510
LWR6		0.5262	0.3163		0.5933
LWR44	0.3173	0.5863		-0.3529	0.4302
LWR18		0.3424	0.4158		0.6355
LWR19			0.6835		0.4468
LWR45				0.4564	0.7567
LWR46		0.3205			0.8223
LWR47	0.5917				0.5388
LWR20				0.6516	0.4899
LWR48				0.7157	0.4577
LWR21		0.5500		-0.4123	0.4794

Loadings represent standardized factor loadings based on Varimax rotation. Loadings <0.3 are blanked.

Table B.3: Exploratory Factor Analysis Models for Right-Wing Radicalism

Variable	Factor 1	Factor 2	Factor 3	Factor 4	Factor 5	Factor 6	Uniqueness
RWR26	0.6734						0.4255
RWR10	0.4576	0.3884		0.5105			0.3493
RWR11	0.6066			0.4138			0.3730
RWR1		0.4924				0.5297	0.3235
RWR25	0.3782	0.5799					0.3699
RWR27	0.4478	0.4200		0.3914		0.3198	0.3393
RWR12	0.6068						0.4911
RWR13	0.7290						0.3617
RWR14	0.6395	0.3380					0.4023
RWR28		0.8011					0.2290
RWR15	0.3876	0.7494					0.2467
RWR2		0.8142					0.2076
RWR16	0.6332						0.4543
RWR3	0.7504						0.3413
RWR4	0.7064	0.3145					0.3740
RWR29	0.6360	0.3021					0.3822
RWR6	0.7632						0.2807
RWR17	0.6173						0.4695
RWR30		0.3380		0.3202			0.6294
RWR31	0.5973	0.3163					0.4629
RWR32	0.6838	0.3234					0.3039
RWR33	0.7464						0.3904
RWR34	0.5868						0.5134
RWR35		0.3433	0.3901	0.4363			0.5143
RWR36		0.3267	0.3552	0.4485			0.4981
RWR37	0.3150			0.4633			0.5379
RWR38	0.6437						0.4722
RWR39	0.7616						0.3769
RWR40					0.8561		0.2513
RWR41					0.8602		0.2559
RWR42	0.4376	0.3919	0.4365				0.4587
RWR43		0.8295					0.2280
RWR44	0.3776	0.7630					0.2554
RWR45	0.7062						0.4122
RWR46	0.6191	0.3201					0.4235
RWR47	0.5221	0.4603					0.4504
RWR48	0.7216	0.3424					0.3204
RWR49						0.7447	0.2930
RWR50			0.7837				0.2933
RWR51	0.3525		0.6672				0.4098
RWR52			0.8115				0.2858
RWR53			0.8119				0.2923
RWR54			0.8158				0.2757
RWR7	0.6943	0.4405					0.2677
RWR55	0.3141	0.6330					0.3530
RWR5	0.3632	0.8010					0.2022
RWR56	0.7085						0.3317
RWR57	0.3485	0.3171		0.6028			0.3541

Loadings represent standardized factor loadings based on Varimax rotation. Loadings <0.3 are blanked.

Table B.4: Exploratory Factor Analysis Models for General Extremism

Variable	Factor 1	Uniqueness
GEX6		0.8121
GEX7	0.7971	0.3646
GEX8		0.7060
GEX9		0.9370
GEX23		0.9187
GEX3	0.7519	0.4346
GEX10	0.6982	0.5125
GEX24		0.6430
GEX11		0.6933
GEX39	0.6190	0.6168
GEX12	0.7081	0.4985
GEX13		0.6987
GEX14		0.8005
GEX16		0.8465
GEX25		0.8220
GEX17		0.8893
GEX26		0.9721
GEX18		0.9443
GEX19	0.6867	0.5284
GEX27	0.7840	0.3853
GEX28	0.7372	0.4566
GEX29	0.7994	0.3610
GEX1	0.7072	0.4998
GEX2	0.6067	0.6319
GEX20	0.7609	0.4210
GEX30	0.6736	0.5462
GEX4	0.7717	0.4044
GEX5	0.6818	0.5351
GEX31	0.6983	0.5124
GEX21	0.7973	0.3644
GEX22	0.7587	0.4243
GEX32		0.6649

Loadings represent standardized factor loadings based on Varimax rotation. Loadings <0.3 are blanked.

C Question Wording

Table C.1: Question Wording of Right-Wing Radicalism Indicators

Variable	Question Wording	Original study	Pre-Test	Main Study
RWR1	We should have the courage to have a strong sense of national consciousness.	Mitte-Studien	x	x
RWR2	The federal republic has become "too foreign" to a dangerous extent due to all the foreigners here.	Mitte-Studien	x	x
RWR3	Jews work more with evil tricks than others in order to get what they want.	Mitte-Studien	x	x
RWR4	Jews simply have something special and peculiar about them and do not really fit in with us.	Mitte-Studien	x	x
RWR5	Foreigners and asylum seekers are the ruin of (country).	Neu (1997)	x	x
RWR6	Actually, (country) are inherently superior to other people.	Mitte-Studien	x	x
RWR7	We should make sure we keep (nationality) pure and prevent nations mixing.	Neu (1997)	x	x
RWR8	The world would be a better place if people from other countries were more like (country).			x
RWR9	Under some circumstances, a nondemocratic government can be preferable.			x
RWR10	(Country) now needs a strong party which embodies the people's community.	Mitte-Studien, Stöss (2006)	x	x
RWR11	We should have a leader who governs (country) with a strong hand for the good of everyone.	Mitte-Studien	x	x
RWR12	If it were not for the Holocaust, Hitler would today be regarded as a great statesman.	Mitte-Studien	x	x
RWR13	The crimes of National Socialism have been greatly exaggerated in the history books.	Mitte-Studien	x	x
RWR14	National Socialism also had its good side.	Mitte-Studien	x	x
RWR15	If there is a shortage of jobs, foreigners should be sent back to their home countries.	Mitte-Studien	x	x
RWR16	Even today, the influence of Jews is still too great.	Mitte-Studien	x	x
RWR17	There is precious and not precious life.	Mitte-Studien	x	x
RWR18	Crimes should be punished more severely.	GMF (2008)	x	x
RWR19	To maintain law and order, we need to be tougher on outsiders and troublemakers.	GMF (2008)	x	x
RWR20	The most important characteristics anyone should have include obedience and respect of superiors.	GMF (2008)	x	x
RWR21	I sometimes feel like an outsider in my own country due to the many Muslims here.	GMF (2004)		x
RWR22	Whites are, rightly so, leading in the world.	GMF (2008)	x	x
RWR23	We need someone again who can provide direction in politics.	Neu (1997)		x
RWR24	Many things done out of patriotism and nationalism have caused more harm than good.			x
RWR25	What our country needs is a tough and energetic enforcement of American interests in the world rather than listening to the interests of foreign countries.	Mitte-Studien	x	
RWR26	In the national interest, a dictatorship is the better form of government under certain circumstances.	Mitte-Studien	x	
RWR27	The primary goal of German policy should be to give Germany the power and authority it deserves.	Mitte-Studien	x	
RWR28	The foreigners only come here to take advantage of our welfare state.	Mitte-Studien	x	
RWR29	Just like in nature, the strongest should always prevail in society.	Mitte-Studien	x	
RWR30	The disputes between the various interest groups are detrimental to the people as a whole.	Stöss (2006)	x	
RWR31	Elections are only good if they do not put leftists in power.	Stöss (2006)	x	
RWR32	Other nations may have achieved important things, but they do not come close to German achievements.	Stöss (2006)	x	
RWR33	We spend too much effort on the care and support of the disabled.	Stöss (2006)	x	

Continued on next page

Table C.1: Question Wording of Right-Wing Radicalism Indicators

Variable	Question Wording	Original study	Pre-Test	Main Study
RWR34	Mentally ill people should be forbidden to have children.	Stöss (2006)	x	
RWR35	Crimes should be punished more severely.	GMF (2008)	x	
RWR36	To preserve law and order, we should be tougher on outsiders and troublemakers.	GMF (2008)	x	
RWR37	Among the most important qualities that someone should have are obedience and respect for the superior.	GMF (2008)	x	
RWR38	Women should once again focus more on the role of wife and mother.	GMF (2008)	x	
RWR39	For a wife, helping her husband with his career should be more important than having a career herself.	GMF (2008)	x	
RWR40	Discrimination against women is still a problem in Germany.	GMF (2008)	x	
RWR41	Current employment policies disadvantage women.	GMF (2008)	x	
RWR42	We take too much notice of failures in our society.	GMF (2008)	x	
RWR43	The many Muslims here sometimes make me feel like a stranger in my own country.	GMF (2008)	x	
RWR44	Muslims should be prohibited from immigrating to Germany.	GMF (2008)	x	
RWR45	The groups that are at the bottom in our society should stay at the bottom.	GMF (2008)	x	
RWR46	There are groups in the population that are worth less than others.	GMF (2008)	x	
RWR47	Aussiedler should be better off than foreigners because they are of German descent.	GMF (2008)	x	
RWR48	Whites are rightly leading the world.	GMF (2008)	x	
RWR49	I am proud to be German.	GMF (2009)	x	
RWR50	Most long-term unemployed people are not really interested in finding a job.	GMF (2011)	x	
RWR51	If you can't find a job after being unemployed for a long time, it's your own fault.	GMF (2011)	x	
RWR52	I find it outrageous when the long-term unemployed make a comfortable life for themselves at the expense of society.	GMF (2011)	x	
RWR53	The long-term unemployed should be required to perform community service.	GMF (2011)	x	
RWR54	The long-term unemployed should only receive money from the state if they are willing to accept any job.	GMF (2004)	x	
RWR55	I would like to see a Germany in which the German people finally have their say again.	Neu (1997)	x	
RWR56	Germans are superior to other peoples.	Neu (1997)	x	
RWR57	There has to be someone again who says where things stand in politics.	Neu (1997)	x	

Table C.2: Question Wording of Left-Wing Radicalism Indicators

Variable	Question Wording	Original study	Pre-Test	Main Study
LWR1	A decent life is only possible under a socialist government.	Schroeder and Deutz-Schroeder (2015)	x	x
LWR2	Capitalism is ruining the world.	Neu (1997)	x	x
LWR3	Fascism shows the true face of capitalism	Schroeder and Deutz-Schroeder (2015)	x	x
LWR4	The (nationality) foreign policy is racist.	Schroeder and Deutz-Schroeder (2015)	x	x
LWR5	The persecution of and spying on left-wing system critics by the state and police is increasing.	Schroeder and Deutz-Schroeder (2015)	x	x
LWR6	National states should be abolished.	Schroeder and Deutz-Schroeder (2015)	x	x
LWR7	The most important businesses need to be nationalised.	Stöss (2006)	x	x
LWR8	The federal army must be equipped with the latest weapons	Schumann (2001)	x	x
LWR9	Capitalism inevitably leads to poverty and hunger	Schroeder and Deutz-Schroeder (2015)	x	x
LWR10	Freedom of expression and the right to demonstrate should not apply to right-wing extremists.	Schroeder and Deutz-Schroeder (2015)	x	x
LWR11	Deeply rooted racism can be seen everywhere in daily life.	Schroeder and Deutz-Schroeder (2015)	x	x
LWR12	(Country) should, in principle, take in everyone who wants to come here	Schroeder and Deutz-Schroeder (2015)	x	x
LWR13	There shouldn't be any (nationality) interests.	Schroeder and Deutz-Schroeder (2015)	x	x
LWR14	Our parliamentary democracy isn't a real democracy as the economic elite decides everything.	Schroeder and Deutz-Schroeder (2015)	x	x
LWR15	A real democracy is only possible without capitalism	Schroeder and Deutz-Schroeder (2015)	x	x
LWR16	Socialism is a good idea which has until now only been put into practice badly	Stöss (2006), Schroeder and Deutz-Schroeder (2015)	x	x
LWR17	The increasing intensification of internal security will lead to a dictatorship.	Schroeder and Deutz-Schroeder (2015)	x	x
LWR18	It should be possible to also use violence to fight Nazis.	Baier and Pfeiffer 2011	x	x
LWR19	Right-wing parties and comradeships should be banned.	Baier and Pfeiffer 2011	x	x
LWR20	In some circumstances a war is necessary to get justice.		x	x
LWR21	The federal army should be vehemently scaled down.		x	x
LWR22	American imperialism is the real danger to world peace.	Stöss (2006)	x	
LWR23	Real democracy is only possible when there is no more capitalism.	Stöss (2006)	x	
LWR24	Only the powerful economic interests benefit from globalization	Stöss (2006)	x	
LWR25	I think it is terrible how the Palestinians are trying to destroy the state of Israel.	GMF (2004)	x	
LWR26	It is unjust for Israel to take land away from the Palestinians.	GMF (2004)	x	
LWR27	Israel is waging a war of extermination against the Palestinians.	GMF (2004)	x	
LWR28	Those who do not act radically cannot realize the true ideals in politics.	Neu (1997)	x	

Continued on next page

Table C.2: Question Wording of Left-Wing Radicalism Indicators

Variable	Question Wording	Original study	Pre-Test	Main Study
LWR29	In politics, there is only true and false. That's why you can't compromise.	Neu (1997)	x	
LWR30	Group and association interests should be subordinated unconditionally to the common good.	Falter et al. (1994-2002)	x	
LWR31	The socialist ideals of society will one day prevail.	Sächsiche Längsschnittstudie SLS-19 (2005)	x	
LWR32	The plundering of the Third World by the capitalist industrialized countries must be stopped	Schroeder and Deutz-Schroeder (2015); Stöss (2006)	x	
LWR33	Capitalism must be overcome in order to abolish the rule of a small minority over the large majority.	Schroeder and Deutz-Schroeder (2015)	x	
LWR34	Capitalism inevitably leads to warfare.	Schroeder and Deutz-Schroeder (2015)	x	
LWR35	Neighborhood and housing modernization in Germany serves only to increase profits.	Schroeder and Deutz-Schroeder (2015)	x	
LWR36	I see the danger of a new fascism in Germany.	Schroeder and Deutz-Schroeder (2015)	x	
LWR37	We find right-wing extremism everywhere in society and not just in small marginalized groups. // Right-wing extremism can be found everywhere in society and not only in small fringe groups.	Schroeder and Deutz-Schroeder (2015)	x	
LWR38	(Parliamentary) democracy and fascism are two sides of capitalism.	Schroeder and Deutz-Schroeder (2015)	x	
LWR39	Reforms will not improve living conditions - we need a revolution.	Schroeder and Deutz-Schroeder (2015)	x	
LWR40	The political center labels its critics as extremists.	Schroeder and Deutz-Schroeder (2015)	x	
LWR41	The equality of all people is more important than the freedom of the individual.	Schroeder and Deutz-Schroeder (2015)	x	
LWR42	German police protect right-wing extremists and fight leftists.	Schroeder and Deutz-Schroeder (2015)	x	
LWR43	The judiciary and the police are instruments of the rulers.	Schroeder and Deutz-Schroeder (2015)	x	
LWR44	In the future, no more euros may be spent on the military.	Baier and Pfeiffer 2011	x	
LWR45	Run-down neighborhoods rich in tradition should be comprehensively renewed, even if this means that some of the existing tenants have to move.		x	
LWR46	Culturally challenged neighborhoods suffering from an economic downturn should not be subject to extensive redevelopment and restructuring under any circumstances.		x	
LWR47	International organizations are taking too much power away from the German government.		x	
LWR48	The use of military forces is sometimes necessary to bring about peace.		x	

Table C.3: Question Wording of General Extremism Indicators

Variable	Question Wording	Original study	Pre-Test	Main Study
GEX1	It is better for government leaders to make decisions without consulting anyone.	Schulz et al. (2018)	x	x
GEX2	People in government must enforce their authority even if it means violating the rights of some citizens.	Schulz et al. (2018)	x	x
GEX3	Under some circumstances, a nondemocratic government can be preferable.	Arab Barometer	x	x
GEX4	A concentration of power in one person guarantees order.	Schulz et al. (2018)	x	x
GEX5	The government should close communication media that are critical.	Schulz et al. (2018)	x	x
GEX6	Despite drawbacks, democracy is the best system of government.	Arab Barometer	x	x
GEX7	A strong non-democratic leader that does not bother with parliament and elections is a good way of governing your country.	Arab Barometer	x	x
GEX8	A rule by experts/technocrats is a good way of governing your country.	Arab Barometer	x	
GEX9	Democracy allows for the government to ensure law and order.	Arab Barometer	x	x
GEX10	(Country) should abolish elections and experts should lead us	Asian Barometer	x	x
GEX11	In important cases, judges should accept the decisions of the executive	Asian Barometer	x	x
GEX12	A totally ethical and moral political leader should be allowed to decide on everything	Asian Barometer	x	x
GEX13	Society becomes chaotic if there are too many different ways of thinking	Asian Barometer	x	x
GEX14	Political leaders can ignore minority groups if they are supported by the majority	Asian Barometer	x	x
GEX15	Political leaders must be able to tolerate those who question their beliefs	Asian Barometer	x	x
GEX16	The media should be able to report news without state censorship	Pew Global Attitudes	x	x
GEX17	People should be able to say what they want without state censorship	Pew Global Attitudes	x	x
GEX18	Opposition parties should be able to operate freely	Pew Global Attitudes	x	x
GEX19	I prefer a non-democratic government that pursues a policy I agree with, rather than a democratically elected government whose policies I oppose		x	x
GEX20	People whose opinions are different than those of the government must be considered its enemies	Schulz et al. (2018)	x	x
GEX21	It is necessary to do away with the democratic form of government if we want to have a decent society.	Ozer and Bertelson (2018)	x	x
GEX22	Just let the rest of the society choose democracy – I, and those who think like me, work to establish up a different system in our own milieu	Ozer and Bertelson (2018)	x	x
GEX23	Democracy is a Western form of government that is not compatible with Islam. (only for Muslims)	Arab Barometer	x	
GEX24	During a crisis, government does not need not obey the law	Asian Barometer	x	
GEX25	Women should have the same rights as men	Pew Global Attitudes	x	
GEX26	Honest elections should be held regularly with a choice of at least two political parties	Pew Global Attitudes	x	
GEX27	I would support a political system governed by a strong authority which makes decisions without considering electoral results or the opinions of the opposition.	Hoffman (2020)	x	
GEX28	I would support a political system governed by religious leaders.	Hoffman (2020)	x	
GEX29	I would support a political system governed by the army.	Hoffman (2020)	x	
GEX30	It is fair that the government does not comply with the law when it thinks it is not necessary	Schulz et al. (2018)	x	
GEX31	If the president does not agree with Congress, he/she should dissolve it	Schulz et al. (2018)	x	
GEX32	It is wrong and immoral to live peacefully side by side with people who don't live the good and correct life.	Ozer and Bertelson (2018)	x	
GEX33	Those who do not act radically cannot realize the true ideals in politics.	Neu (1997)	x	

Continued on next page

Table C.3: Question Wording of General Extremism Indicators

Variable	Question Wording	Original study	Pre-Test	Main Study
GEX34	In politics, there is only true and false. That's why you can't compromise.	Neu (1997)	x	
GEX35	In the national interest, a dictatorship is the better form of government under certain circumstances.	Mitte-Studien	x	
GEX36	(Country) now needs a strong party which embodies the people's community.	Mitte-Studien, Stöss (2006)	x	x
GEX37	The most important characteristics anyone should have include obedience and respect of superiors.	GMF (2008)	x	x
GEX38	There has to be someone again who says where things stand in politics.	Neu (1997)	x	
GEX39	Government can't accomplish great things if always checked by legislature	Asian Barometer	x	

References

Altemeyer, Bob and Bruce Hunsberger (2004). “A Revised Religious Fundamentalism Scale: The Short and Sweet of It”. In: *International Journal for the Psychology of Religion* 14 (1), 47–54.

Baier, Dirk and Christian Pfeiffer (2011). *Jugendliche als Opfer und Täter von Gewalt in Berlin.* Forschungsbericht 114. Hannover: Kriminologisches Forschungsinstitut Niedersachsen e.V. (KFN).

Bruder, Martin, Peter Haffke, Nick Neave, Nina Nouripanah, and Roland Imhoff (2013). “Measuring Individual Differences in Generic Beliefs in Conspiracy Theories Across Cultures: Conspiracy Mentality Questionnaire”. In: *Frontiers in Psychology* 4.

Cherney, Adrian, Jennifer Bell, Ellen Leslie, Lorraine Cherney, and Lorraine Mazerolle (2018). *Countering Violent Extremism Evaluation Indicator Document.* University of Queensland: Australian, New Zealand Counter-Terrorism Committee, National Countering Violent Extremism Evaluation Framework, and Guide.

Decker, Oliver, Johannes Kiess, and Elmar Brähler, eds. (2022). *The Dynamics of Right-Wing Extremism within German Society: Escape into Authoritarianism.* Routledge studies in fascism and the far right. Abingdon, Oxon; New York, NY: Routledge.

Falter, Jürgen W., Oscar W. Gabriel, Hans Rattinger, and Klaus Schmitt (2012). *Political Attitudes, Political Participation and Voting Behavior in Reunified Germany (Panel 1994-2002).* ZA4301, Data file Version 1.1.0. Cologne: GESIS Data Archive.

Glazier, Rebecca A. (2015). “Bridging Religion and Politics: The Impact of Providential Religious Beliefs on Political Activity”. In: *Politics and Religion* 8 (3), 458–487.

Heitmeyer, Wilhelm, Andreas Zick, Steffen Kühnel, Peter Schmidt, Ulrich Wagner, and Jürgen Mansel (2013a). *Group-oriented animosity against people (GMF-Survey 2004).* Version Number: 2.0.0 Type: dataset.

Heitmeyer, Wilhelm, Andreas Zick, Steffen Kühnel, Peter Schmidt, Ulrich Wagner, Jürgen Mansel, and Jost Reinecke (2013b). *Group-oriented animosity against people (GMF-Survey 2008).* Version Number: 1.0.0 Type: dataset.

— (2013c). *Group-oriented animosity against people (GMF-Survey 2011).* Version Number: 1.0.0 Type: dataset.

Hoffman, Michael (2020). “Religion, Sectarianism, and Democracy: Theory and Evidence from Lebanon”. In: *Political Behavior* 42 (4), 1169–1200.

Isenhardt, Anna, Maria Kamenowski, Patrik Manzoni, Sandrine Haymoz, Cédric Jacot, and Dirk Baier (2021). “Identity Diffusion and Extremist Attitudes in Adolescence”. In: *Frontiers in Psychology* 12, 711466.

Jungkunz, Sebastian (2022). *The Nature and Origins of Political Extremism In Germany and Beyond.* Cham: Palgrave Macmillan.

— (2023). “The Measurement of Left-Wing Extremist Attitudes”. In: *The Palgrave Handbook of Left-wing Extremism.* Ed. by José Pedro Zúquete. Vol. 1. Cham: Palgrave Macmillan, 141–156.

Kalmoe, Nathan P. and Lilliana Mason (2019). “Lethal Mass Partisanship: Prevalence, Correlates, & Electoral Contingencies”. In: *Prepared for presentation at the January 2019 NCAPSA American Politics Meeting.*

Muthén, Linda K. and Bengt O. Muthén (1998/2017). *Mplus User's Guide*. 8th ed. Los Angeles: Muthén & Muthén.

Neu, Viola (2009). *Rechts- und Linksextremismus in Deutschland. Wahlverhalten und Einstellungen*. Sankt Augustin, Berlin: Konrad-Adenauer-Stiftung.

Ozer, Simon and Preben Bertelsen (Dec. 2018). "Capturing violent radicalization: Developing and validating scales measuring central aspects of radicalization". In: *Scandinavian Journal of Psychology* 59 (6), 653–660.

Schroeder, Klaus and Monika Deutz-Schroeder (2015). *Gegen Staat und Kapital - für die Revolution! Linksextremismus in Deutschland - eine empirische Studie*. Frankfurt: Peter Lang.

Schulz, Wolfram, John Ainley, Cristián Cox D., and Tim Friedman (2018). *Young people's views of government, peaceful coexistence, and diversity in five Latin American countries: IEA International Civic and Citizenship Education Study 2016 Latin American Report*. Cham: Springer.

Schumann, Siegfried (2001). *Persönlichkeitsbedingte Einstellungen zu Parteien*. München, Wien: Oldenbourg.

Statistisches Bundesamt (Destatis) (2022). *Internationale Bildungsindikatoren im Ländervergleich. Ausgabe 2022 – Tabellenband*. Wiesbaden: Statistisches Bundesamt.

Stöss, Richard, Michael Fichter, Joachim Kreis, and Bodo Zeuner (2004). *Projekt "Gewerkschaften und Rechtsextremismus": Abschlussbericht*. Berlin: Hans-Böckler-Stiftung, Otto-Brenner-Stiftung.

UNECE Statistical Database (2023). *Educational attainment by level of education, age and sex*. URL: `https://w3.unece.org/PXWeb2015/pxweb/en/STAT/STAT__30-GE__04-EducatAndcommunicat/003_en_GEECEduAttainmSPN_r.px/`.

United Nations (2023). *Demographic Yearbook 2022*. New York: United Nations Department of Economic and Social Affairs.
