# Supplementary material for: Measuring political radicalism and extremism in surveys: Three new scales
Source: PLoS One. 2024 May 8;19(5):e0300661. doi: 10.1371/journal.pone.0300661 (PMC11078353; doi:10.1371/journal.pone.0300661)
Supplement: Attachment — Submitted filename: reveiw_takano_ed.pdf [file pone.0300661.s002.pdf]

The present study developed three novel scales, applicable across Western European nations, to examine right-wing, left-wing, and general extremist attitudes (RWR, LWR, GEX). Utilizing data from Germany, Great Britain, and the Netherlands, the researchers examined the validity of these scales by evaluating the psychometric properties of the derived indices, including their internal consistency and capacity to differentiate among strongly extremist individuals. Furthermore, the researchers investigated the relationships among the scales and various constructs associated with extremist attitudes to establish external and construct validity. The findings substantiate the high validity of these three scales, which are applicable across Western European nations.

Overall, the manuscript is well-written, and the results are clearly presented. I have a few comments regarding relatively minor issues, which are explained below.

1. (p. 2, lines 13–23) The tone of the sentences in this section could be modulated to present a more balanced perspective. It is important to note that several established standardized measures, such as the right-wing authoritarianism scale, attempt to assess related constructs, even though their primary focus may not be right- or left-wing extremism.

2. The authors repeatedly refer to the Appendix, but the precise location of the relevant information within the Appendix remains unclear.

3. (pp. 9–10, lines 208-241) Clarification is needed regarding the distinction between a scalar model and a partial scalar model. Additionally, is the context of ΔRMSEA and ΔCFI significant regarding the difference between these two types of models?

4. (p. 11, lines 261–269) I want to make sure I understand this portion correctly. Is the claim based on the right-skewed peak in the information curves and the fact that SE curves do not have high values on the right side in Figure 2?

5. Descriptive summary statistics are provided in the supplementary material. However, the authors should include these details in the main text because information such as participant age and gender is vital to the readers.

6. The study should present the correlations between RWR, LWR, and GEX. This would enable a discussion of the claim that GEX is non-compensatory, based on various findings in the study.

7. In Table 1, please consider adding the mean and standard deviation for each item within each scale.

8. The study should acknowledge its limitations. Every research study has constraints and acknowledging them helps temper the claims made. Please list two to four limitations.
