# Supplementary material for: Measuring political radicalism and extremism in surveys: Three new scales
Source: PLoS One. 2024 May 8;19(5):e0300661. doi: 10.1371/journal.pone.0300661 (PMC11078353; doi:10.1371/journal.pone.0300661)
Supplement: Attachment — Submitted filename: response_letter.pdf [file pone.0300661.s003.pdf]

Response Letter for "Measuring political radicalism and extremism in surveys: three new scales"

Dear editor and reviewers,

thank you very much for giving us the opportunity to revise and resubmit our manuscript.

We are grateful for the positive feedback as well as the constructive comments and suggestions. The editor and reviewers have offered thoughtful and well taken points to improve the manuscript. We have taken these comments very seriously and tried our best to accommodate all suggestions. In this memo, we outline in detail how we have addressed each of them.

We are optimistic and hope that the thoroughly revised manuscript now meets your demands.

Reviewer 1

COMMENT 1.1

This is a very good, insightful and neat study. I strongly recommend it to be published subject to minor revisions. Below are some suggestions.

"right- and left-wing radicalism" ... "understand people who take drastic or far-reaching positions on issues such as nationalism or anti-imperialism" (p.2): To me that is not a particularly precise definition. First I would try to define it without the "or" and leave out the "drastic" (what is drastic?). Second, I wonder whether one could refine what the authors understand by "far-reaching".

RESPONSE 1.1

Thank you very much! We revised the definition and framed it more in line with the

existing literature in the field. We now define it as: "The second dimension then differentiates between right- and left-wing radicalism by which we understand people who take far-reaching but often one-sided positions on political issues (e.g., on nationalism or anti-imperialism) by advocating fundamental socio-political change (Bötticher 2017; Jungkunz 2022; Pugh 2009)."

In fact, it is often difficult to rely on pre-existing definitions, as they may include the word "extreme" in it. For instance, the Cambridge dictionary defines radicalism as "the belief that there should be great or *extreme* social or political change" (emphasis added, `https://dictionary.cambridge.org/dictionary/english/radicalism`). Similarly, the Oxford dictionary defines it as "Advocating thorough or far-reaching political or social reform; representing or supporting an *extreme* section of a party" (emphasis added, `https://www.oed.com/dictionary/radical_adj?tab=meaning_and_use#27278303`). "Far-reaching" is not necessarily considered as an absolute term but refers rather to the relative difference between radical propositions and the political standpoint of mainstream public opinion and politics.

COMMENT 1.2

The authors mention a few other concepts that should be differentiated from extremism such as fanaticism, terrorism. Generally, the focus is on convergent validity and less on discriminant validity albeit the authors write that "our scales are able to discriminate well between radicalism and actual extremism". (p.14). To me it's a limitation of the current paper that the discussion and empirical differentiation from other concepts gets not spotlight. Hence, I would add this to the conclusion as a venue for future research and point the way where to go and to look.

RESPONSE 1.2

We are thankful for the comment and we added this as future research idea to the conclusion of the paper.

COMMENT 1.3

“Finally, we treat political extremism as a non-compensatory concept [24–26] that considers someone as extremist only when he or she holds strong left- or right-wing radical attitudes like socialism or social-Darwinism and anti-democratic attitudes at the same time.”: The authors’ definition implies that someone CANNOT be considered an extremist if they only hold strong left- or right-wing radical attitudes without the anti-democratic component. This has quite far-reaching consequences (if everyone would really follow their advice). While the authors cite others I would like them to add a paragraph that more extensively explains this decision in the present paper. What are the pros and cons of adding the anti-democratic component to the definition and why do you do it?

Could there be extremists that hold radical beliefs but do not hold anti-democratic attitudes? For example, could there be an individual that holds extreme environmental views and engages in non-violent protest but does not necessarily oppose democratic principles. I guess following the authors this person would not be an extremist. A discussion of interesting edge cases would be extremely helpful.

And, how do environmental or religious extremism fit into the author’s definition? Someone who is radically anti-abortion but pro-democratic ist not an extremist I guess. Could these other forms of extremism be included? (religious extremism is mentioned in the appendix page 2).

RESPONSE 1.3

Your assumption that “authors’ definition implies that someone CANNOT be considered an extremist if they only hold strong left- or right-wing radical attitudes without the anti-democratic component”, is correct. We do this exactly to remove much of the confusion in the existing literature, political discourse, and in the media, and to create more precision between terms. This allows us to distinguish individuals that hold (merely) radical attitudes e.g., in terms of xenophobia or anti-capitalism, but who still stay in bounds of the democratic constitutional state from others who clearly object democracy and are in favor of e.g. autocratic regimes that work against the principles of democracy, i.e. extremists.

We agree that this might, at first sight, be a strong assumption, but it gives researchers much greater precision between concepts. Such practice has been proposed before, e.g. by Goertz Goertz (2006, 2020), and be discussed with practical application by others ((e.g., Jungkunz, Fahey, and Hino 2021; Wuttke, Schimpf, and Schoen 2020). Furthermore, this definition is used by public authorities as well, e.g. the German Federal Office for the Protection of the Constitution. Importantly, this does not mean that we disregard (mere) radicalism, but rather it allows us to e.g. identify factors that drive people towards holding radical attitudes and other factors that makes them to lose their support for democracy. Any compensatory approach would disregard this important information and we would not be able to distinguish between the two concepts.

Based on these remarks, the person described by the reviewer ("an individual that holds extreme environmental views and engages in non-violent protest but does not necessarily oppose democratic principles") would be considered *radical*. If the person then leaves the bounds of democracy, he or she is considered extremist.

Environmental and religious extremism fit in this definition as well. Environmental extremism could probably be considered a subcategory of left-wing extremism. Furthermore and according to Altemeyer (1996), religious extremism (REX) shares characteristics with political extremism and is defined as the "belief that there is one set of religious teachings that clearly contains the fundamental, basic, intrinsic, essential, inerrant truth about humanity and deity" and "that this essential truth is fundamentally opposed by forces of evil which must be vigorously fought" (Altemeyer and Hunsberger 1992, 118). The fundamental equality between human beings and their individual freedom is also negated (Brandt and Reyna 2010). Moreover, there is a clear anti-liberal element when it is argued that religious rules should have priority over secular laws (Koopmans 2015, 35). We would, however, consider religious extremism as a separate, distinct concept which is why we do not discuss it extensively in the text.

COMMENT 1.4

In measurement we can make a fundamental distinction between formative and reflective indicators/measurement. It would be helpful if the authors could point out which approach they are following with which method, e.g., see Coltman et al. (2008).

RESPONSE 1.4

We apologize for being somewhat imprecise. We follow the reflective approach which we now state in the paper in footnote 3.

COMMENT 1.5

"The survey was administered in each country's primary language and is representative in terms of sex, age, and education" (p.7): A comparison of the survey data to census statistics would be great (for all three countries). Right now the reader has no way to check that statement. In addition, the authors should outline how representativity was reached (quota sampling? weighting?). In principle, quota sampling could be stated as a limitation in the conclusion for transparency reasons.

RESPONSE 1.5

We added comparison tables with official statistics from census data to the appendix. Quotas were realized through stratified random sampling. We specified this in footnote 2.

COMMENT 1.6

There is no discussion of response rates and not analysis of missing data. Both would be good to have in the appendix. The naniar R package is quite useful to visualize missingness in the dataset.

RESPONSE 1.6

We now provide more information about the sample and the respective response rate in footnote 1. We also followed your advice and added a figure about missing data using the nainar R package to the appendix (Figure A.14) and we refer to it in the main text.

COMMENT 1.7

"Finally, none of the scales holds particularly bad loadings, as the lowest standardized loading is 0.460 for LWR." (p.22): What are acceptable cutoffs here?

RESPONSE 1.7

There are no particular cutoffs for hard categorizations of standardized factor loadings. We would rather interpret them in line with common practices of interpreting correlation coefficients. However, Comrey and Lee 1992, for instance, regarded standardized factor loading lower than 0.3 as bad. We now refer to their work in the paper.

COMMENT 1.8

Table 1: Right now there is no discussion of the single items that go into the scales. However, I imagine that a lot of discussions have happened prior to the selection of those items. Ideally, the authors would add a bit more background (even if only in the appendix) on the selection of those items.

RESPONSE 1.8

We agree that some more information might be necessary. We now highlighted in the text that "in general, we selected the items with the highest loadings in exploratory factor analyses. However, in cases where items achieved similar or very close factor loadings, we opted for items that added more conceptual breadth to the scale. Furthermore, we made sure to include items that include no country-specific information and which can be understood in the same way across countries". In the appendix A and B we present in more detail how we selected the original list of up to 180 items and how we cut them down to around 25 items per scale for the main survey in various pretests. The entire list of items can be found in Appendix C.

COMMENT 1.9

Table B1: Why not show the shares of different educational levels?

RESPONSE 1.9

We added a more fine-grained specification of educational levels to Table B.1

COMMENT 1.10

Figure 2: If you are allowed to print color you could combine the plots into one (with three different colors) which would facilitate the comparison that you describe in the text.

RESPONSE 1.10

We sticked with common practices that present IRT models side by side. The advantage in this format is that the space of the curves within the plot area is maximized. Collapsing all models into one figure is difficult because the y-axes are somewhat different. Since information values are not interpreted in absolute terms but rather in comparison to the standard errors (i.e. judging where information decreases and imprecision increases), we opted to keep it this way.

COMMENT 1.11

Table 4: This is pooled across all 3 countries. However, it probably also makes sense to show this data unpooled for the three different countries in the appendix.

RESPONSE 1.11

We apologize for being imprecise here and we thank you for pointing this out. We already included these figures in the previous version in the manuscript (Supplementary Material Tables A.14–A.16). Unfortunately, we did not mention them in the text which is why they might have gone missing. We added a note to the text to make it clearer.

COMMENT 1.12

Aggregation & Multiplicative index (see below): I am not completely sold on this idea and got a bit carried away. On the one hand we somehow need to combine the two dimensions (radicalism + general extremism) to follow the author's definition. I tried to do a very simple simulation in R with the code attached for the multiplicative index (which might be wrong). Underlying the same value of the multiplicative index there may still be variation in the underlying dimensions. Hence, I would recommend the authors to alert readers to the potential of such variation. For some values of the multiplicative index the two sub

dimensions may diverge quite strongly. This could matter once we relate the index to other dimensions. In addition, it would be nice to see such distributions for the empirical data that was actually collected by the authors in the appendix. The theoretical range of the index may not reflect the empirical one.

RESPONSE 1.12

We agree that aggregation methods are often subject to arbitrary decision-making on part of the researchers. Therefore, we wanted to stick with existing methods that have been used before by other researchers. Since it might be argued that other operationalizations result in different conclusions, we now provide the results from two other aggregation methods: a minimum value version and an additive version. The minimum value version is created based on taking the minimum value across the respective radicalism scale and the general extremism scale. The additive version is created based on taking the mean value across the respective radicalism scale and the general extremism scale. We discuss the results and limitations of these methods in footnote 8 (see also Figures A.10 and A.11 in the Supplementary Material). We further provide correlations between different aggregation methods for reference in Figures A.12 and A.13 in the Supplementary Material.

COMMENT 1.13

Future research: The authors mention social desirability as one challenge. However, I imagine that there are other venues of research that might spring from this paper (i.e., what would the authors like to do in the future) that should be discussed in the conclusion. In part, these should be linked to the limitations the present study shares with others, e.g., restricted to few countries, few time points, potentially biased sample representativity of the sample with regard to political orientations etc.

RESPONSE 1.13

We agree with that and we provide more research avenues in the discussion of the paper (see also RESPONSE 2.8)

COMMENT 1.14

Finally, I found a data availability statement but I wonder about the embargo period. I would expect the authors to publish raw data & code files to reproduce all analyses and graphs upon publication. The raw data can be a subset of the larger survey I guess.

RESPONSE 1.14

We apologize for the confusion. We will provide a replication data set including the main variables and code in case of acceptance. These will be stored in the corresponding author's Harvard Dataverse. Our previous statement alluded to making all data (current and still to collect data of the entire research project) available after an embargo period. It is currently planned to harmonize all data sets and upload them as a larger data file to the GESIS archive (`www.gesis.org`) to make it available for the research community.

REVIEWER 2

COMMENT 2.1

Overall, the manuscript is well-written, and the results are clearly presented. I have a few comments regarding relatively minor issues, which are explained below.

1. (p. 2, lines 13–23) The tone of the sentences in this section could be modulated to present a more balanced perspective. It is important to note that several established standardized measures, such as the right-wing authoritarianism scale, attempt to assess related constructs, even though their primary focus may not be right- or left-wing extremism.

RESPONSE 2.1

We are thankful for the suggestions and we now refer to other related concepts like right- (and left-)wing authoritarianism (Altemeyer 1996; Conway et al. 2018; Hiel, Duriez, and Kossowska 2006), authoritarian personality (Adorno et al. 1950; Oesterreich 2005), or social dominance orientation (Sidanius and Pratto 1999) that have been developed over time, but where it is unclear how they are related to political extremism.

COMMENT 2.2

2. The authors repeatedly refer to the Appendix, but the precise location of the relevant information within the Appendix remains unclear.

RESPONSE 2.2

We apologize for being imprecise. We now added the exact locations of the respective tables and figures to the main manuscript.

COMMENT 2.3

3. (pp. 9–10, lines 208-241) Clarification is needed regarding the distinction between a scalar model and a partial scalar model. Additionally, is the context of ΔRMSEA and ΔCFI significant regarding the difference between these two types of models?

RESPONSE 2.3

We now elaborate more on partial scalar invariance in the respective paragraph and we better connect the Table with the results from ΔRMSEA and ΔCFI. Furthermore, we also refer to additional work by Putnick and Bornstein (2016) on measurement invariance testing.

COMMENT 2.4

4. (p. 11, lines 261–269) I want to make sure I understand this portion correctly. Is the claim based on the right-skewed peak in the information curves and the fact that SE curves do not have high values on the right side in Figure 2?

RESPONSE 2.4

This is correct. In all three figures, the information curve is skewed slightly to the right, meaning that the scale has more information in higher levels of the scale (i.e. it can distinguish respondents better at the upper end of the scale, hence the SEs are also lower). At the lower end, they can distinguish respondents somewhat less well. In practice, this means that the scale is less suited to distinguish non-radical and non-extremist respondents from somewhat (in the sense of very slightly) radical or extremist ones. Since this is, however, not our main focus and the scales are able to capture respondents at the upper ends (radical, extremist) well, we concluded that the scales are well suited for radicalism and extremism research.

COMMENT 2.5

5. Descriptive summary statistics are provided in the supplementary material. However, the authors should include these details in the main text because information such as participant age and gender is vital to the readers.

RESPONSE 2.5

We added the information in footnote 2.

COMMENT 2.6

6. The study should present the correlations between RWR, LWR, and GEX. This would

enable a discussion of the claim that GEX is non-compensatory, based on various findings in the study.

RESPONSE 2.6

We now provide the correlations by country in Figures A.12 and A.13 in the Supplementary Material.

COMMENT 2.7

7. In Table 1, please consider adding the mean and standard deviation for each item within each scale.

RESPONSE 2.7

Table 1 is intended to only include the question wording for the reader. However, we agree that more information on the items might be helpful which is why we now provided them additionally in Figures A.1 to A.3.

COMMENT 2.8

8. The study should acknowledge its limitations. Every research study has constraints and acknowledging them helps temper the claims made. Please list two to four limitations.

RESPONSE 2.8

We agree that the limitations should have been more detailed. We now elaborate more on the selectivity of the countries and the wider applicability, the need for more empirical work on the distinction of radicalism and extremism from related concepts like fanaticism and terrorism, and potential social desirability bias.

References

Adorno, Theodor W., Else Frenkel-Brunswick, Daniel J. Levinson, and R. Nevitt Sanford, eds. (1950). *The Authoritarian Personality.* New York: Harper & Brothers.

Altemeyer, Bob (1996). *The Authoritarian Specter.* Cambridge: Harvard University Press.

Altemeyer, Bob and Bruce Hunsberger (1992). "Authoritarianism, Religious Fundamentalism, Quest, and Prejudice". In: *International Journal for the Psychology of Religion* 2 (2), 113–133.

Bötticher, Astrid (2017). "Towards Academic Consensus Definitions of Radicalism and Extremism". In: *Perspectives on Terrorism* 11 (4), 73–77.

Brandt, Mark J. and Christine Reyna (2010). "The Role of Prejudice and the Need for Closure in Religious Fundamentalism". In: *Personality and Social Psychology Bulletin* 36 (5), 715–725.

Comrey, Andrew Laurence and Howard B. Lee (1992). *A First Course in Factor Analysis.* 2nd ed. New York: Psychology Press.

Conway, Lucian Gideon, Shannon C. Houck, Laura Janelle Gornick, and Meredith A. Repke (2018). "Finding the Loch Ness Monster: Left-Wing Authoritarianism in the United States". In: *Political Psychology* 39 (5), 1049–1067.

Goertz, Gary (2006). *Social Science Concepts.* Princeton: Princeton University Press.

— (2020). *Social Science Concepts and Measurement: New and Completely Revised Edition.* Princeton: Princeton University Press.

Hiel, Alain van, Bart Duriez, and Malgorzata Kossowska (2006). "The Presence of Left-Wing Authoritarianism in Western Europe and Its Relationship with Conservative Ideology". In: *Political Psychology* 27 (5), 769–793.

Jungkunz, Sebastian (2022). *The Nature and Origins of Political Extremism In Germany and Beyond.* Cham: Palgrave Macmillan.

Jungkunz, Sebastian, Robert A. Fahey, and Airo Hino (2021). "How populist attitudes scales fail to capture support for populists in power". In: *PLoS ONE* 16 (12), e0261658.

Koopmans, Ruud (2015). "Religious Fundamentalism and Hostility against Out-groups: A Comparison of Muslims and Christians in Western Europe". In: *Journal of Ethnic and Migration Studies* 41 (1), 33–57.

Oesterreich, Detlef (2005). "Flight into Security: A New Approach and Measure of the Authoritarian Personality". In: *Political Psychology* 26 (2), 275–297.

Pugh, Jonathan (2009). *What is Radical Politics Today?* New York: Palgrave Macmillan.

Putnick, Diane L. and Marc H. Bornstein (2016). "Measurement invariance conventions and reporting: The state of the art and future directions for psychological research". In: *Developmental Review* 41, 71–90.

Sidanius, Jim and Felicia Pratto (1999). *Social Dominance: An Intergroup Theory of Social Hierarchy and Oppression*. Cambridge University Press.

Wuttke, Alexander, Christian Schimpf, and Harald Schoen (2020). "When the Whole Is Greater than the Sum of Its Parts: On the Conceptualization and Measurement of Populist Attitudes and Other Multidimensional Constructs". In: *American Political Science Review* 114 (2), 356–374.
